# Ischemic injury triggers a protective microglial phenotype in models of Aβ pathology

**DOI:** 10.1186/s12974-026-03897-x

**Published:** 2026-06-09

**Authors:** Michael Candlish, Jan Hofmann, Desirée Brösamle, Annika Haessler, Murphy DeMeglio, Angelos Skodras, Georgi Tushev, Eloah S. De Biasi, Stefan Günther, René Wiegandt, Heidi Theis, Elena De Domenico, Nina Sofia Hermann, Peter Breunig, Christina Sauerland, K. Peter R. Nilsson, Marc D. Beyer, Mario Looso, Maike Windbergs, Sigrun Roeber, Jochen Herms, Jonas J. Neher, Andreas G. Chiocchetti, Jasmin K. Hefendehl

**Affiliations:** 1 Neurovascular Disorders, Institute of Cell Biology and Neuroscience, Biologicum, Goethe University Frankfurt, Max-von-Laue Str. 13, Frankfurt am Main, Germany; 2 Department of Cellular Neurology, Hertie Institute for Clinical Brain Research, University of Tübingen, Tübingen, Germany; 3 Biomedical Center (BMC), Biochemistry, Faculty of Medicine, LMU Munich, Munich, Germany; 4 Neuroimmunology and Neurodegenerative Diseases, German Center for Neurodegenerative Diseases (DZNE), Munich, Germany; 5 Munich Cluster for Systems Neurology (SyNergy), Munich, Germany; 6 Institute of Pharmaceutical Technology, Goethe University Frankfurt, Max-von- Laue-Str. 9, Frankfurt am Main, Germany; 7 Max Planck Institute for Brain Research, Max-von-Laue-Str. 4, Frankfurt am Main, Germany; 8 Max Planck Institute for Heart and Lung Research, Member of the German Center for Lung Research (DZL), Member of the Cardio-Pulmonary Institute (CPI), Bad Nauheim, Germany; 9 Platform for Single Cell Genomics and Epigenomics (PRECISE) at the German Center for Neurodegenerative Diseases (DZNE), Bonn, Germany; 10 Immunogenomics & Neurodegeneration, German Center for Neurodegenerative Diseases (DZNE), Bonn, Germany; 11 Department of Physics, Chemistry and Biology, Linköping University, Linköping, SE-581 83 Sweden; 12 Center of Neuropathology and Prion Research, Faculty of Medicine, LMU Munich, Munich, Germany; 13 Center for Neuropathology, Ludwig-Maximilians-University Munich, Munich, Germany; 14 Department of Child and Adolescent Psychiatry, Psychosomatics and Psychotherapy, University Hospital, Goethe University Frankfurt, Frankfurt am Main, Germany

**Keywords:** Alzheimer’s disease, Stroke, Microglia, Co-morbidity

## Abstract

Microglia are highly plastic cells that are capable of integrating subsequent insults. As the majority of Alzheimer’s Disease (AD) patients also show cerebrovascular pathology, we here aimed to dissect the interactions between AD and ischemic brain injury on the microglial response to amyloid beta (Aβ) pathology. Unexpectedly, ischemic stroke in the context of cerebral β-amyloidosis drives the emergence of a neuroprotective microglial phenotype characterized by an ApoE-enriched transcriptional state and enhanced lipid handling. These microglia promote the rapid formation of highly compact Aβ plaques that are relatively inert and strikingly reminiscent of those observed in cognitively resilient AD patients. Our findings thus reveal that the microglial response to Aβ pathology is not a fixed trajectory toward dysfunction, but retains a capacity for beneficial reprogramming when engaged by the appropriate stimulus. Beyond characterizing this comorbid state, our data identify specific molecular pathways, centered on ApoE, complement activation, and lysosomal processing, that may be amenable to therapeutic targeting to promote protective microglial function in AD.

**Supplementary Information:**

The online version contains supplementary material available at 10.1186/s12974-026-03897-x.

## Introduction

Alzheimer’s disease (AD) and cerebrovascular disease remain leading causes of mortality and morbidity. Notably, a significant proportion of AD cases exhibit signs of cerebrovascular pathology upon post-mortem examination [1]. Consequently, recent studies have aimed to investigate potential links between vascular dysfunction and subsequent cognitive impairment [2–4], as it is essential to analyze the interaction of co-morbid disease states. Microglia, the resident immune cells of the brain, are critically involved in a range of neurodegenerative diseases [5, 6]. They are pivotal in their response to stroke and AD but how they respond to the combined presence of both diseases remains uncertain, given their highly dynamic nature. Microglia possess the capacity to engage in both detrimental and reparative processes [7] in the context of neurological disease depending on both the pathology and disease stage. In AD, along with other neurodegenerative diseases, microglia enter a disease-associated (DAM) phenotype [8]. In mouse models of cerebral β-amyloidosis, DAM cluster around amyloid beta (Aβ) plaques, engage in Aβ phagocytosis [9] and act as a physical barrier that restricts the expansion of the immature, neurotoxic Aβ halo surrounding mature (less neurotoxic) Aβ plaque cores [10]. As the pathology advances, there is an increased abundance of immature Aβ within the brain parenchyma, coupled with a decline in the formation of mature, dense-cored Aβ plaques and an increase of more toxic soluble prefibrillar Aβ [11]. This suggests a progressive alteration in the protective function of microglia over time [12]. Conversely, in stroke, microglia contribute to acute neuroinflammation and exacerbate neuronal loss, but also promote neuroprotection and post-ischemic repair. Importantly, the balance between these effects varies over the recovery period [13–15]. Taken together, the distinct pathological milieus of AD pathology and ischemic stroke in a co-morbid condition hold the potential to modulate microglial response, with unknown influence on AD progression.

Here, we analyze the intricate interplay between ischemic stroke and AD pathology by utilizing the APPPS1 mouse model in conjunction with ischemic stroke. Using this murine co-morbidity model, we identify a microglia-dependent accumulation of Aβ plaques within the vulnerable peri-infarct region as rapidly as three weeks after stroke. This phenomenon occurred despite a well-established age/pathology-dependent reduction in the rate of *de novo* Aβ plaque formation [11]. Notably, newly-formed peri-infarct Aβ plaques exhibit a relatively benign nature, and are encapsulated by an overabundance of phagocytotic microglia. Using single-cell RNA sequencing (scRNA-seq) and spatial transcriptomics, we demonstrate that ischemic stroke in the context of cerebral β-amyloidosis does not simply amplify the canonical disease-associated microglial response, but instead induces a broader transcriptional remodeling of the microglial compartment. Cross-dataset integration identified ApoE as the central molecular driver of this reprogramming, consistently upregulated across all relevant microglial clusters and peri-infarct Aβ plaque-associated spatial spots - converging on a microglial state characterized by enhanced lipid handling, complement activation, and lysosomal processing. Taken together, our findings demonstrate that the pathological microenvironment created by ischemic stroke in the context of Aβ pathology triggers a functional switch in microglia that promotes the formation of highly compact Aβ plaques strikingly similar to those observed in patients resilient to the detrimental effects of AD pathology [16].

## Results

### Ischemic stroke triggers the accumulation of Aβ plaques proximal to the infarct border

**Fig. 1:**
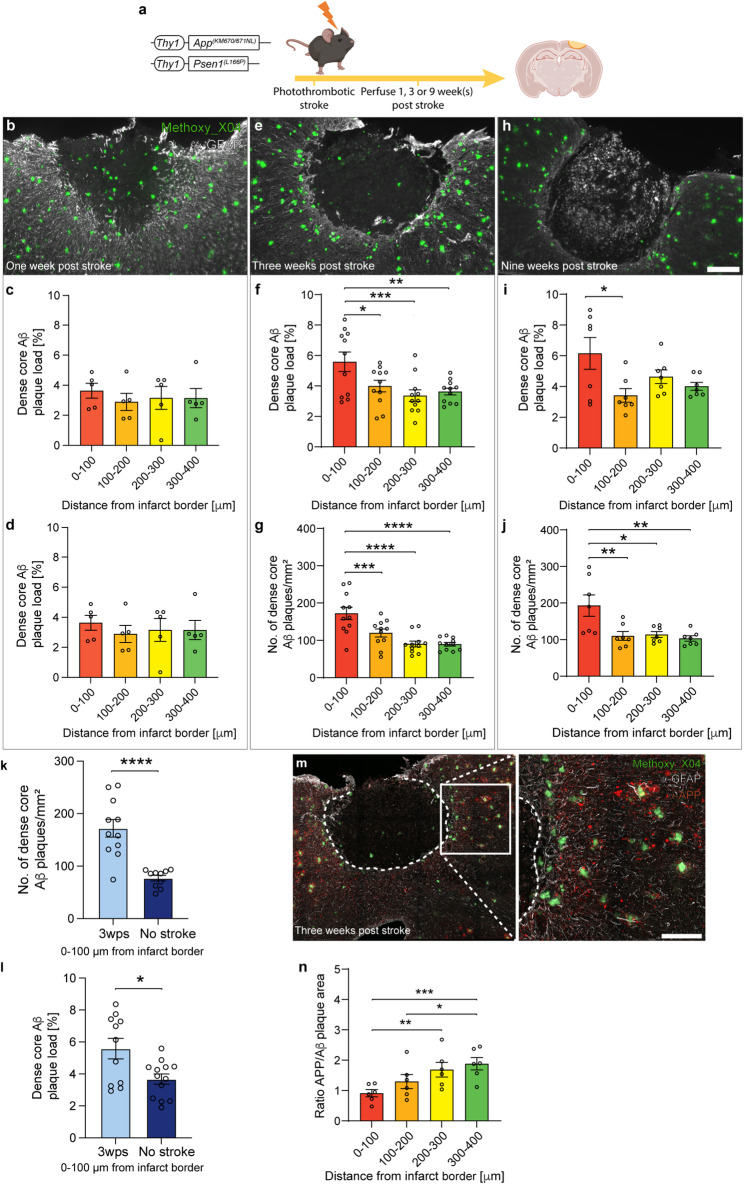
Ischemic stroke drives an accumulation of Aβ plaques in the peri-infarct zone. **a** Experimental strategy to model ischemic stroke in a mouse model of cerebral β-amyloidosis (APPPS1 mice). **b** Representative images of APPPS1 mouse brain sections one, **e** three and **h** nine weeks post stroke. Scale bar = 200 μm. **c** No significant difference in dense-core Aβ plaque load or **d** the number of dense core Aβ plaques in the peri-infarct region (*n* = 5 mice; 3 male, 2 females) at one week post stroke. **f** Significantly higher dense-core Aβ plaque load as well as= **g** the number of dense core Aβ plaques were found in close proximity to the infarct border (*n* = 11 mice; 6 males, 5 females) at three weeks post stroke. **i** Increased dense-core Aβ plaque load and **j** number of dense-core Aβ plaques persisted in close proximity to the infarct border up until at least nine weeks post stroke (*n* = 7 mice, 3 males, 4 females). **k** The number of dense core Aβ plaques /mm^2^ (*n* = 11 three weeks post stroke mice, 6 males, 5 females, *n* = 10, 5 males, 5 females no stroke APPPS1 mice) and **l** the dense core Aβ plaque load is significantly higher 0–100 μm from the infarct border three weeks post stroke compared to APPPS1 mice without stroke. (*n* = 11 three weeks post stroke mice, 6 males, 5 females, *n* = 13 no stroke APPPS1 mice, 7 males, 6 females). **m** Representative image of an APPPS1 mouse brain section three weeks post stroke with APP immunolabelling to visualize dystrophic neurites. Scale bar = 50 μm (inset). **n** Significantly less Aβ-plaque associated axonal dystrophy was present in close proximity to the infarct border (*n* = 6 mice, 3 males, 3 females). Dense-core Aβ plaques (**b**, **e**, **h**, **m**) are visible in green (labelled with Methoxy_X04), glial scar (**b**, **e**, **h**, **m**) is visible in white (GFAP immunoreactivity). Dystrophic neurites **m** are visible in red (amyloid precursor protein (APP) immunoreactivity). Repeated-measures one-way ANOVA with Tukey’s multiple comparison test for **c**, **d**, **f**, **g**, **i**, and **n**. Unpaired two-tailed Student’s t-test for **k**, **l**. * = *p* < 0.05, ** = *p* < 0.01, *** = *p* < 0.001, **** = *p* < 0.0001. For full statistical details, see Supplementary Table 2

To establish the impact of ischemic stroke on Aβ deposition and distribution within both the stroke core and surrounding tissue, we employed a photothrombotic stroke model in (5–18 months old) APPPS1 mice (a well-established and aggressive model of Aβ deposition [17] that develops cerebral β-amyloidosis already at six weeks of age). Aβ plaque distribution was subsequently analyzed at one, three- and nine-weeks post stroke (Fig. 1a). Additionally, we performed whole-brain tissue clearing using iDISCO at one- and three-weeks post stroke (Extended Data Video 1 and 2) in tandem with histological approaches. At one week post stroke we found no significant differences in mature dense-core (Methoxy-X04^+^) Aβ plaque load (percentage of brain area occupied by Aβ plaques) or the number of dense-core Aβ plaques in the peri-infarct tissue, suggesting no short-term effect on the examined pathological hallmarks (Fig. 1b-d, Extended Data Video 1). However, at three weeks post stroke, we observed a readily visible reduction in the number of Aβ plaques within the infarct core. In contrast, we detected a significant increase in both dense-core Aβ plaque load and number of dense-core Aβ plaques in the tissue adjacent to the infarct border (Fig. 1e-g, Extended Data Video 2). This phenomenon persisted up until nine weeks post stroke (Fig. 1h-j). Comparing the number of dense-core Aβ plaques within the 0–100 μm region around the infarct border at three weeks post stroke to control APPPS1 animals, we found that both the number of Aβ plaques and Aβ dense-core plaque load were significantly higher in the peri-infarct zone (Fig. 1k, l). To determine whether this phenomenon might be exclusive to the APPPS1 mouse model, we analyzed brains from a second model with cerebral β-amyloidosis, APP23 mice, three weeks after stroke (Extended Data Fig. 1a). The APP23 mouse model features a higher ratio of Aβ40/42, considerably slower onset of Aβ deposition and kinetics as well as differences in Aβ plaque morphology compared to APPPS1 mice [17, 18]. Strikingly, we found a similar accumulation of Aβ plaques in the peri-infarct region, suggesting that this phenomenon is independent of the model of cerebral β-amyloidosis (Extended Data Fig. 1b-d). It is important to note, that the formation rate of *de novo* dense-core Aβ plaques starts to decrease from approximately five months of age in APPPS1 mice without stroke (Extended Data Fig. 2a-m). Rather, there is a somewhat slow increase in the size of existing dense-core Aβ plaques [11]. To visualize immature Aβ species [19], we used the luminescent conjugated oligothiophene (LCO) hFTAA (the emission spectra of which varies depending upon the Aβ structure to which they are bound [20]). Using this technique, we are able to infer Aβ conformation with spatial resolution. Using this dye, we identified an accumulation of toxic immature Aβ species (Extended Data Fig. 2a-m) over time in APPPS1 mice without stroke. As all animals used in the present study were five months of age or older, our data thus indicate that ischemic stroke radically alters Aβ plaque accumulation and deposition kinetics within the vulnerable peri-infarct region as early as three weeks post stroke, resulting in the formation of new Aβ plaques and an apparently accelerated aggregation rate.

### Peri-infarct Aβ plaques are structurally distinct and comparatively benign

We noticed that the peri-infarct Aβ plaques appeared morphologically distinct from the previously described Aβ plaques in APPPS1 mice, presenting with a more well-defined edge as opposed to a transition to immature neurotoxic Aβ halo as is typically seen around Aβ plaques in APPPS1 mice [21]. As more fibrillar (less compact) Aβ plaques have been linked to increased axonal dystrophy [10], we hypothesized that peri-infarct Aβ plaques might display a lower neurotoxicity compared to “classical” Aβ plaques (non-stroke associated Aβ plaques). We therefore assessed the accumulation of the axonal dystrophy markers amyloid precursor protein (APP) [22] as well as LAMP1 (Extended data Fig. 3a-b) in axons surrounding peri-infarct Aβ plaques. Indeed, we found significantly less dystrophic neurites surrounding Aβ plaques in close proximity to the infarct border from three weeks post stroke onwards (Fig. 1m, n, Extended Data Fig. 2n-s), suggesting that the peri-infarct Aβ plaques are comparatively benign.

**Fig. 2:**
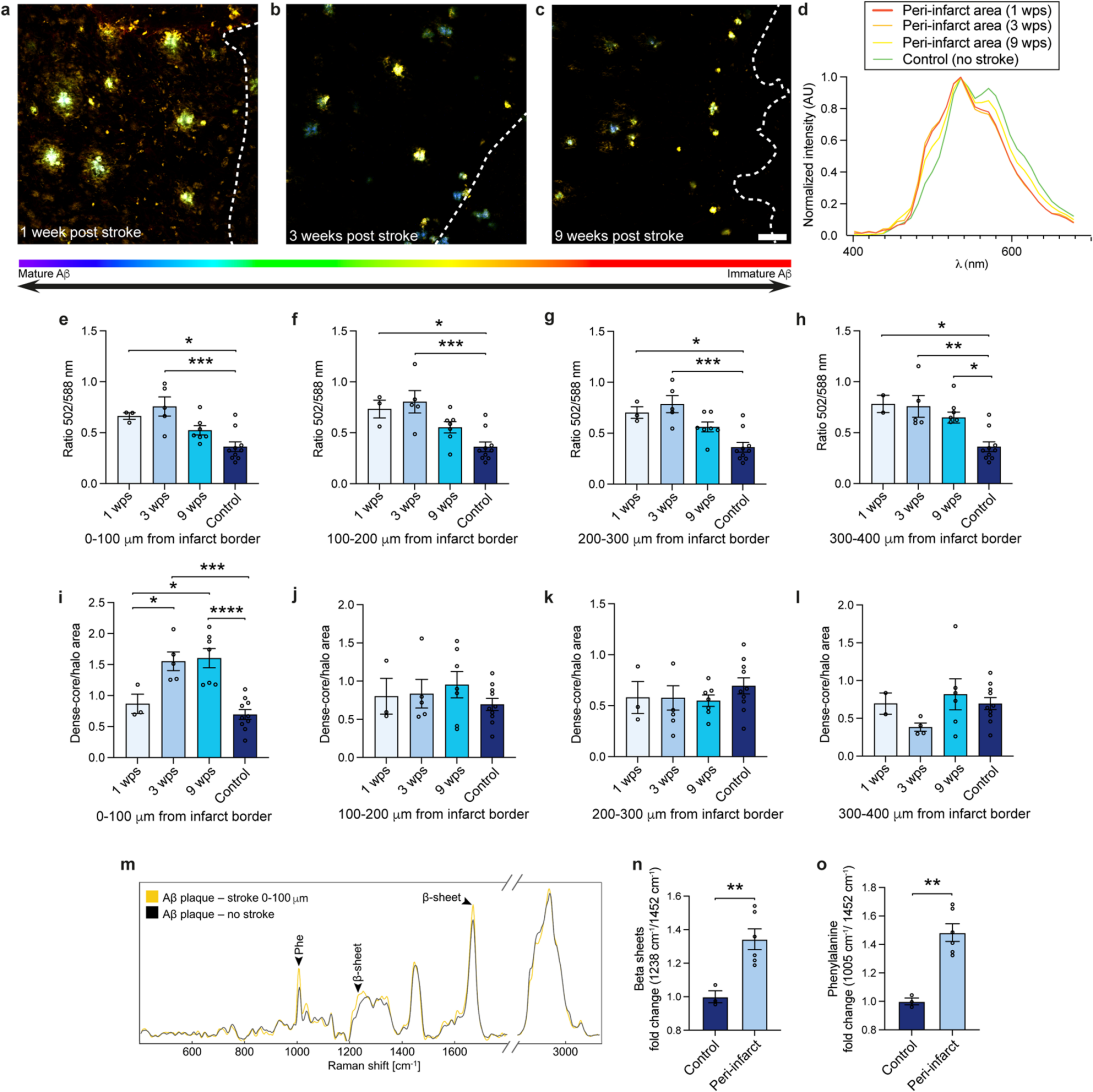
Peri-infarct Aβ plaques feature less neurotoxic immature Aβ halo and are more dense than typical Aβ plaques. **a-c** False-color coding of spectral scan images of APPPS1 brain sections stained with hFTAA and qFTAA taken at one **a**, three **b** and nine **c** weeks post stroke (dashed lines demarcate the border of the infarct core, scale bar = 50 μm). Note the absence of immature Aβ halo as well as non-Aβ plaque associated immature Aβ deposits from three weeks post stroke onwards. **d** Representative normalized emission spectra traces of Aβ plaques in the peri-infarct area one-, three- and nine-weeks post stroke and without stroke. Note the general blue (i.e. left) shift of emission spectra in stroke compared to no stroke indicating increased density. **e** Dense core Aβ plaque to immature Aβ halo ratio was significantly increased compared to no stroke (and one-week post stroke) from three weeks post stroke onwards in the region 0–100 μm from the infarct border f,g,h but not distally (n = 2–10 mice; one week post stroke 2 male, 1 females, three weeks post stroke 3 males 2 females, 9 weeks post stroke 3 males, 4 females). **i** qFTAA/hFTAA spectral ratio at 502 nm and 588 nm (respectively) of peri-infarct Aβ plaques at one, three and nine weeks post stroke and with no stroke in APPPS1 mice (n = 2–9 mice; one week post stroke 2 males 1 female, three weeks post stroke 3 males 2 females, 9 weeks post stroke 3 males 4 females). A significantly higher qFTAA/hFTAA ratio was found consistently at one and three weeks post stroke compared to control (no stroke) at 0–100 μm,** j** 100–200 μm, **k **200–300 μm and **l** 300–400 μm from the infarct border. **m** Averaged Raman spectra of peri-infarct Aβ plaques and Aβ plaques from APPPS1 mice without stroke. Markings indicate characteristic peaks of β-sheets and phenylalanine. **n** Peak ratio analysis of Raman spectra indicating β-sheets and o phenylalanine (n = 3 mice three weeks post stroke, two Aβ plaques per Sect. (1 male, 2 females), n = 3 mice no stroke APPPS1 controls, one Aβ plaque per Sect. (2 males, 1 female). Repeated-measures one-way ANOVA with Tukey’s multiple comparison test **e-l**. Two-tailed unpaired t-test **j**, **k** * = *p* < 0.05, ** = *p* < 0.01, *** = *p* < 0.001, **** = *p* < 0.0001. For full statistical details, see Supplementary Table 2

To further interrogate the composition of the Aβ plaques in the peri-infarct area, we took advantage of the unique spectral properties of LCOs by using a combination of qFTAA and hFTAA, which have previously been utilized to resolve Aβ plaque maturation in AD mouse models [20, 23]. By analyzing their emission spectra, the conformation of LCO-bound Aβ can be quantified [24]. To this end, we performed spectral scans on Aβ plaques labelled with qFTAA (which binds mature, compacted Aβ) [20, 25] and hFTAA (which binds immature/prefibrillar Aβ) [20, 24] one-, three- and nine-weeks post stroke (Fig. 2a-c). In the 0–400 μm region from the infarct border, we found that Aβ plaques were not only composed of more mature Aβ (as determined by the increased qFTAA to hFTAA ratio) as early as one-week post stroke but also surprisingly exhibited even greater compaction than Aβ plaques from APPPS1 mice without stroke (Fig. 2d-h). Accordingly, we also found a substantial reduction in the neurotoxic hFTAA positive Aβ halo surrounding Aβ plaques within the 0–100 μm perimeter around the infarct core starting at three weeks post-stroke and persisting until at least nine weeks post-stroke (Fig. 2i-l). Additionally, we observed a distinct loss of non-Aβ plaque associated small fibrillar Aβ deposits within the peri-infarct region (Fig. 2a-c). Given that Aβ can also be detected within neurons, we analyzed the intra to extra-neuronal distribution via surface reconstructions of the individual imaged components. We found that in APPPS1 mice, the vast majority of hFTAA-labeled Aβ is found extra-neuronally (Extended Data Fig. 3c-d).

Next, to substantiate our findings we analyzed the compositional alterations in peri-infarct Aβ plaques using confocal Raman microscopy. As a label-free technique, it solely relies on the inelastic scattering of photons by molecular bonds, enabling the acquisition of spatially resolved specific molecular signatures. Consistent with our previous findings, we observed a significant increase in β-sheets as well as phenylalanine (a major component of the peptide primary and secondary structure, thus another indicator of density) within the peri-infarct Aβ plaques compared to typical Aβ plaques (Fig. 2m-o); confirming that ischemic stroke drives the generation of highly compact Aβ plaques in the peri-infarct region.

Thus, our findings reveal dynamic changes in Aβ plaque pathology within the peri-infarct region induced by ischemic stroke, manifesting as heightened accumulation of mature Aβ plaques between one- and three-weeks post-stroke. This coincides with a discernible reduction in non-Aβ plaque associated neurotoxic immature Aβ species. These peri-infarct Aβ plaques exhibit a strikingly dense phenotype that exceeds the compaction of typical Aβ plaques. Furthermore, they feature a diminished fibrillar halo and crucially, induce less Aβ plaque-associated axonal dystrophy compared to typical Aβ plaques.

### Microglial encapsulation of peri-infarct Aβ plaques is augmented after ischemic stroke

**Fig. 3:**
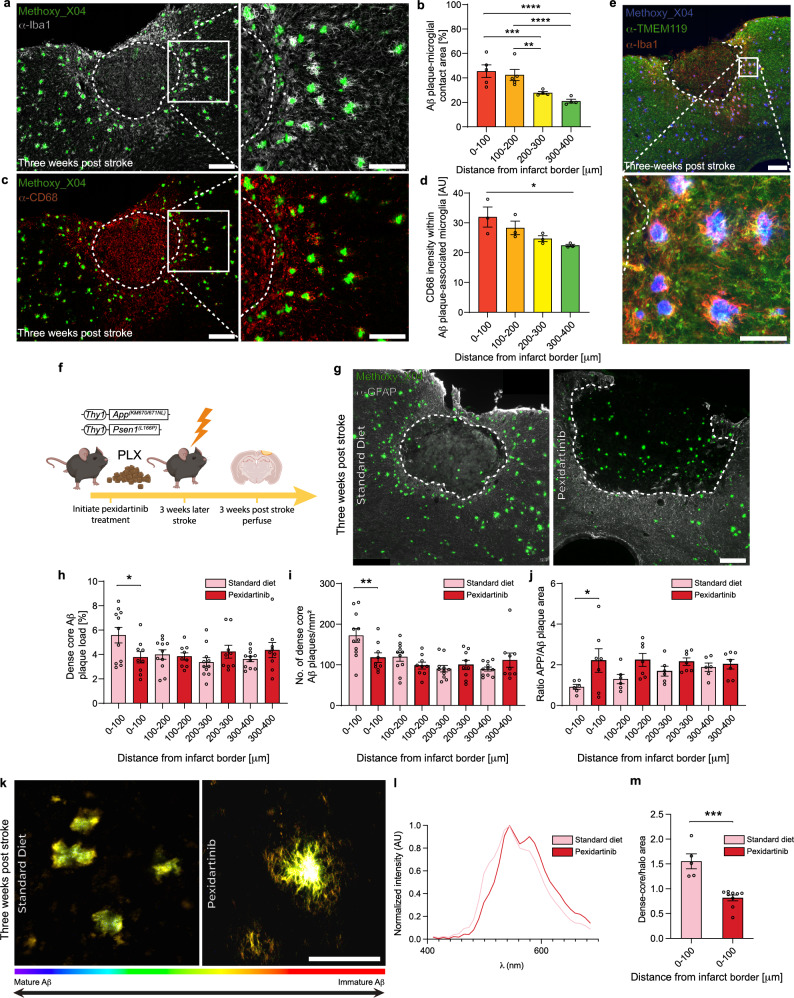
Microglia drive the formation of relatively benign Aβ plaques. **a** Representative image of an APPPS1 mouse brain section three-weeks post stroke with microglia (Iba1^+^ cells) visible in white and dense-core Aβ plaques (labelled with Methoxy_X04) in green. Scale bar = 200 μm, inset 100 μm. **b** Aβ plaque/microglial contact area (i.e. surface-to-surface contact area) is significantly higher in close proximity to the infarct border (*n* = 4–5 mice, 3 males, 2 females). **c** Representative image of an APPPS1 mouse brain section three-weeks post stroke with the phagocytosis marker CD68^+^ cells visible in red and dense-core Aβ plaques (labelled with Methoxy_X04) in green. Scale bar = 200 μm, inset 100 μm. **d** Significantly higher CD68 intensity was detected in Aβ plaque-associated microglia in close proximity to the infarct border (*n* = 3 mice, 1 male, 2 females). **e** Representative image of an APPPS1 mouse brain section three-weeks post stroke with microglia (Iba1^+^ cells) visible in red, the microglia-specific marker TMEM119 visible in green, and dense-core Aβ plaques (labelled with Methoxy_X04) in blue. Scale bar = 200 μm, inset 100 μm. **f** Experimental strategy to deplete microglia prior to ischemic stroke. **g** Representative image of an APPPS1 mouse brain section three-weeks post stroke after (right) microglial depletion with pexidartinib (PLX) and on standard diet (control). Note the lack of Aβ plaque accumulation in the peri-infarct area. Glial scar (GFAP immunoreactivity) is visible in white and dense-core Aβ plaques (labelled with Methoxy_X04) in green. Scale bar = 200 μm. **h** Pexidartinib treatment abolished the increase in dense-core Aβ plaque load and **i** the increased number of dense core Aβ plaques (*n* = 11 standard diet mice, 6 males, 5 females, *n* = 9 pexidartinib treated mice, 5 males, 4 females) **j** Pexidartinib treatment results in significantly more Aβ plaque-associated dystrophic neurites proximal to the infarct border (*n* = 6 standard diet mice, 3 males, 3 females, *n* = 7 pexidartinib treated mice, 5 males, 2 females). **k** False-color coding of spectral scan images of APPPS1 brain sections stained with hFTAA and qFTAA taken at three weeks post stroke following six weeks of pexidartinib treatment (dashed lines demarcate the border of the infarct core, scale bar = 50 μm). **l** Representative normalized emission spectra traces of Aβ plaques in the peri-infarct area at three weeks post stroke with and without pexidartinib treatment. Note the general red (i.e. right) shift of emission spectra resulting from pexidartinib treatment indicating decreased density. **m** Dense core Aβ plaque to immature Aβ halo ratio was significantly decreased after pexidartinib three weeks post stroke in the region 0–100 μm from the infarct border (*n* = 5 standard diet mice, 3 males, 2 females, *n* = 9 pexidartinib treated mice, 4 females, 5 males). Repeated-measures one-way ANOVA with Tukey’s multiple comparison test **b**, **c**. Ordinary one-way ANOVA with Šidák’s multiple comparison test **h**, **i **, **j**, two-tailed Students t-test **m**. * = *p* < 0.05, ** = *p* < 0.01, *** = *p* < 0.001, **** = *p* < 0.0001. For full statistical details, see Supplementary Table 2

To further study the underlying cause for the observed shifts in Aβ plaque deposition and aggregation kinetics, we next focused on microglia. They are of particular interest due to their putative role in Aβ plaque generation and compaction [26], as well as their crucial role in restricting the spread of the toxic immature Aβ halo [10]. In our co-morbidity model, we observed a notable increase not only in the Iba1^+^ area surrounding peri-infarct Aβ plaques near the infarct border (Extended Data Fig. 4a, b), but also a significantly increased microglia/Aβ plaque contact area on proximal peri-infarct Aβ plaques (Fig. 3a, b). Additionally, using PU.1 immunoreactivity to label microglial nuclei, we found that the number of microglia surrounding peri-infarct Aβ plaques is higher proximal to the infarct core (Extended Data Fig. 4c, d). Furthermore, Aβ plaque-associated microglia proximal to the infarct border featured significantly higher CD68 intensity (a lysosomal marker expressed in phagocytotic microglia) (Fig. 3c, d). These observations suggest that following ischemic stroke microglia mount an augmented phagocytic response towards Aβ. Notably, Iba1^+^ cells surrounding the peri-infarct Aβ plaques were TMEM119^+^, thus confirming their microglial identity [27] (Fig. 3e).

To provide mechanistic evidence for the role of microglia in increased peri-infarct Aβ plaque formation, we next depleted microglia using the colony-stimulating factor 1 receptor (CSF1R) antagonist pexidartinib (Fig. 3f). Consistent with previous studies, we attained a significant, yet incomplete, reduction in microglia-occupied area, as DAM [8] no longer require CSF1R activation for their survival [26, 28] (Extended Data Fig. 4e-f). Remarkably, we found that pexidartinib treatment diminished peri-infarct Aβ plaque formation (Fig. 3g, h,i), indicating a requirement of this process for CSF1R-dependent microglia. Likewise, we found that peri-infarct Aβ plaques were less dense after microglial ablation and featured a lower dense-core Aβ plaque to halo ratio, reminiscent of typical Aβ plaques (Fig. 3k-m, Extended Data Fig. 4g). Crucially, axonal dystrophy was significantly higher around the Aβ plaques adjacent to the infarct core in pexidartinib-treated mice, further consolidating the importance of microglia in the described process (Fig. 3j, Extended Data Fig. 4h). Importantly, by analyzing axon density via neurofilament M labelling in brain sections from mice treated with either pexidartinib or standard diet, we found that there were no significant differences between both groups. These findings indicate that higher axonal dystrophy in pexidartinib treated mice is not a result of increased axonal survival after stroke compared to mice with an intact microglial population (Extended data Fig. 5a-b).

Thus, we show that CSF1R-dependent microglia play an essential role in the formation of inert peri-infarct Aβ plaques after stroke. It is worth noting however that the ablation of microglia may have indirect effects on Aβ plaque compaction by diminishing the clearance of myelin and cellular debris [29].

### Augmentation of *Apoe* expression is a defining feature of microglial stroke response in comorbidity

**Fig. 4:**
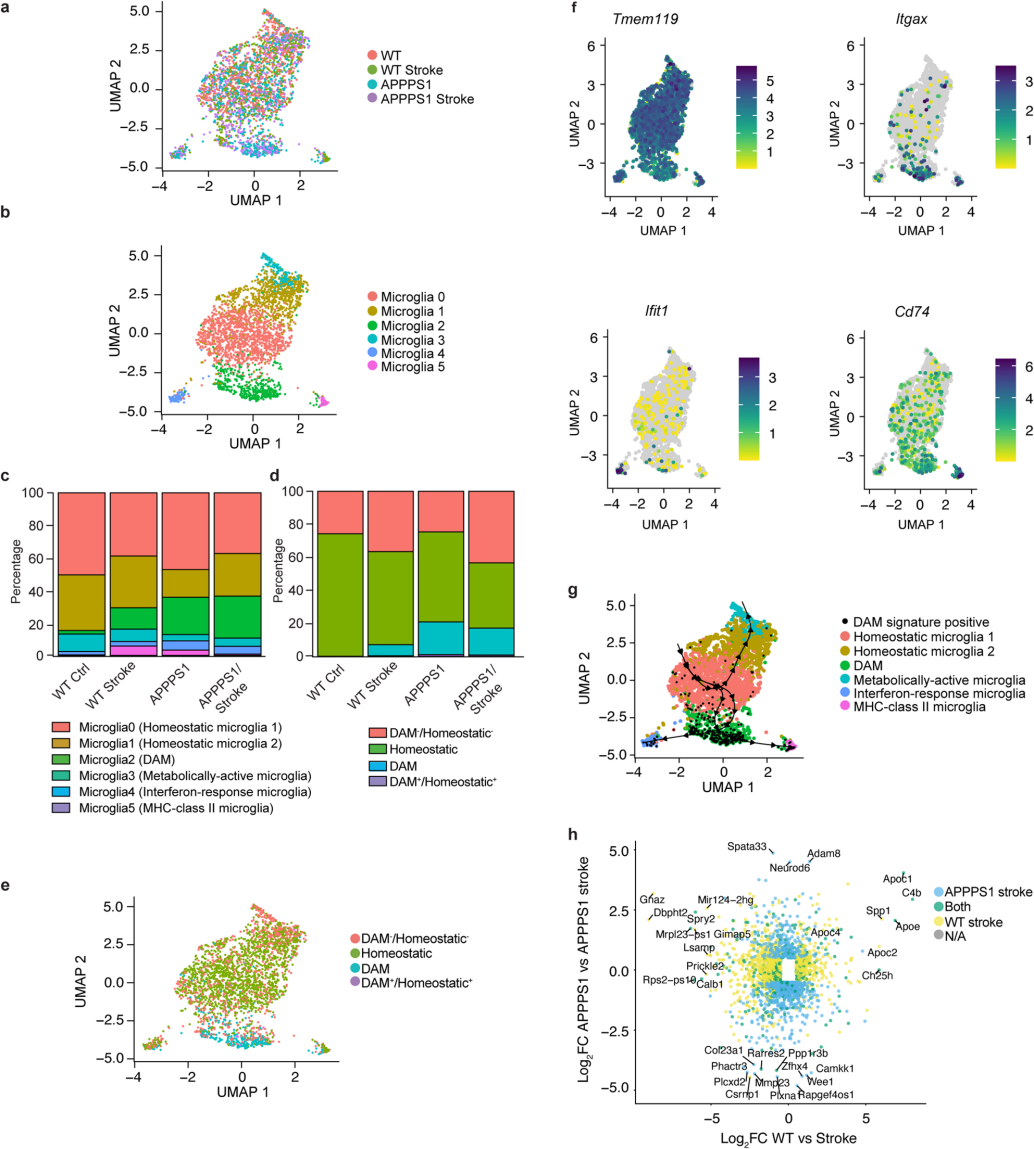
scRNA-seq reveals an expansion of microglia classified as neither DAM nor homeostatic microglia after stroke. **a** Uniform manifold approximation and projection (UMAP) plots of microglia from WT, WT stroke, APPPS1 and APPPS1 stroke mice. **b** UMAP plot of microglial clusters identified in WT, WT stroke, APPPS1 and APPPS1 stroke mice. **c** Stacked bar chart indicating the relative proportion of each microglial cluster in each condition. **d** Stacked bar chart indicating the relative proportion of microglia assigned as homeostatic, DAM, as having features of both DAM and homeostatic (DAM^+^/Homeostatic^+^) or being identified as neither DAM or homeostatic (DAM^−^/Homeostatic^−^). **e** UMAP plots of microglia indicating which microglia have been assigned to each microglia class as described in **d**. **f **UMAP plots illustrating the expression levels of genes associated with homeostatic microglia (Tmem119), DAM (Itgax), interferon-response microglia (Ifit1) and MHC-class II microglia (Cd74). **g** Trajectory analysis indicating transition from (1) homeostatic microglia 1, through homeostatic microglia 2 to the metabolically active cluster, (2) homeostatic microglia 1 through DAM to MHC-class II microglia and (3) homeostatic microglia 1 through DAM to IRM. **h** Scatter plot showing differentially expressed genes in microglia from WT, APPPS1, WT stroke and APPPS1 ^+^ stroke mice. (For scRNAseq experiments, *n = 6* wildtype mice (3 females, 3 male), *n = 5* wildtype stroke (3 females, 2 males), *n = 5* APPPS1 (3 females, 2 males), APPPS1 stroke *n = 5 *(2 females, 3 males). For additional scRNAseq data, see Extended Data Table 1. For full statistical details, see Supplementary Table 2

We next analyzed the molecular signature of the microglia driving the formation of relatively benign Aβ plaques in our co-morbid model by analyzing their transcriptomic profiles. To this end we performed micro-dissections of brain tissue containing the infarct core along with the surrounding peri-infarct tissue and performed fluorescence-activated cell sorting (FACS) for microglia (excluding macrophages by gating for CD11b^high^ and CD45^low^ cells, as described previously [30] (Extended Data Fig. 6a, b). To attain high quality transcriptomic data from the micro-dissected infarct and peri-infarct tissue, which limited the number of isolated cells, we used Smart-seq2 to delineate transcriptomic alterations in stroke-associated microglia in the presence of cerebral-β-amyloidosis. Using this approach, we attained a highly enriched sort of microglia with only few non-microglial cells detected, which were subsequently removed prior to further analysis (Extended data Fig. 6c, d). Even though changes in expression levels were detected, all remaining cells used for subsequent analysis expressed typical microglial marker genes (i.e. *TMEM119*,* P2ry12*,* Cx3Cr1*,* Selplg*; Extended Data Fig. 6e-j, Extended Data Fig. 7). We performed scRNA-seq (Fig. 4a) on microglia sorted from four different conditions: (a) APPPS1 mice three weeks post-stroke (referred to as APPPS1 + stroke), (b) APPPS1 mice without stroke, (c) WT mice three weeks post-stroke (referred to as WT+stroke) and (d) WT mice without stroke. When comparing microglia from all conditions using unsupervised clustering, we identified a total of six clusters (Fig. 4b). In WT mice, as expected, the vast majority of microglia expressed homeostatic markers such as *P2ry12*,* Tmem119* and *Cx3cr1* (microglia clusters 0 (50.4%) and 1 (34.2%)) (Fig. 4a-c, f), as previously described [31] (hence we refer to these clusters as homeostatic microglia 1 and 2, respectively). Conversely, in APPPS1 mice, a substantial microglia cluster (microglia 2) expressed genes such as *Tyrobp*,* Itgax*,* Apoe* and *Clec7a*, consistent with DAM (Fig. 4a-c, f). This population increased from 2.18% in WT mice to 22.9% of the total microglial population in APPPS1 mice. Additionally, the presence of cerebral β-amyloidosis resulted in the expansion of microglial cluster 4 which was enriched for interferon response associated genes such as *Ifit1*, *Ifit2*, *Ifit3* and *Irf7* (hence referred to as interferon-response microglia) (Fig. 4a-c, f). In WT stroke, Microglia 5 - an MHC class II antigen-presenting cluster - was substantially expanded from 0.36% in WT mice, to 5.81% (the largest in any condition tested) in WT stroke. This cluster was characterised by upregulation of *H2-Eb1*,* H2-Aa*,* H2-Ab1* and *Cd74*, canonical MHC class II marker genes (Fig. 4a-c, f). Lastly, we identified a small cluster of microglia (microglia 3) expressing a gene signature suggesting metabolic activity such as *Lipe*, *Or8b44*, *Acnat1* and GPCR-associated gene expression that was most abundant in WT microglia (10.8% in WT, 7.65% in WT stroke), somewhat depleted in APPPS1 mice (3.88% in APPPS1), yet partially restored in APPPS1 stroke mice (5.02%) (Figs. 4a-c and 6g (*Lipe* UMAP)).

To attain an overview of the transcriptomic identity of these clusters, we split the cell populations into either homeostatic, DAM^+^/homeostatic^+^ (featuring markers of both homeostatic microglia and DAM), DAM or neither signatures (DAM^−^/homeostatic^−^, Fig. 4d, e). The vast majority of microglia in WT were enriched for the homeostatic signature (74.2%) with a smaller population determined to be neither DAM nor homeostatic (25.8%). As expected, in APPPS1 a DAM signature was detected in 19.9% of microglia, whereas 54.5% of microglia were classified as homeostatic. The proportion of microglia identified as neither was similar to that of WT mice (24.6%). Intriguingly however, in both WT stroke and APPPS1 + stroke, there was an increase in the proportion of microglia that were neither DAM nor homeostatic (36.5% in WT stroke, 43.4% in APPPS1 stroke) (Fig. 4d, e).

To delineate the transition from homeostatic microglia 1 to the different activation states, we used pseudotime analysis (Fig. 4g). The homeostatic cluster with the highest expression of typical markers for homeostasis (homeostatic microglia 1), was set as the point of origin in this analysis under the assumption that any stimulus-induced phenotype likely arises from these cells. The first trajectory bridged between the two homeostatic microglial clusters prior to terminating in the metabolically active microglial cluster and was characterized by the expression of genes such as *Lipe*, *Or8b44* and *Acnat1* (Extended data Fig. 8a). The second trajectory passes through DAM before terminating within the MHC class II antigen-presenting cluster (characterized by expression of *H2-Ab1*, *H2-Aa* and *Cd74*, Fig. 4g, Extended Data Fig. 8b). The third trajectory transversed homeostatic microglia 1 to DAM (Fig. 4g) and, as expected, was characterized by the upregulation of hallmark DAM-associated genes, including *Cst7*, *Apoe* and *Clec7a*, then terminating within the interferon response microglia cluster (characterized by genes such as *Ifit3*, *Ifit3b* and *Oasl2*, Extended Data Fig. 8c.) These findings are consistent with our data demonstrating that selective ablation of CSF1R-dependent microglia, but not DAM, with pexidartinib abolished the formation of highly dense peri-infarct Aβ plaques.

To characterize the transcriptional nature of this shift across conditions, we performed a cross-condition differential expression analysis comparing log_2_ fold changes in APPPS1 + stroke versus APPPS1 against those in WT stroke versus WT (Fig. 4h). This revealed a subset of genes consistently upregulated across both stroke conditions, prominently including apolipoprotein family members (*Apoe*, *Apoc1*, *Apoc2*) and complement component *C4b*, indicating a shared stroke-driven lipid-metabolic and complement response irrespective of Aβ status. Notably, several genes were specifically upregulated in APPPS1 + stroke but not in WT stroke, pointing to a condition-specific transcriptional program that emerges uniquely at the intersection of ischemic injury and cerebral β-amyloidosis. Hence, these data demonstrate that stroke – whether in APPPS1 or wildtype mice – results in an expansion of microglia that are defined neither as homeostatic or DAM, suggesting that transcriptional changes within existing clusters may be responsible for augmented Aβ plaque compaction after stroke.

**Fig. 5:**
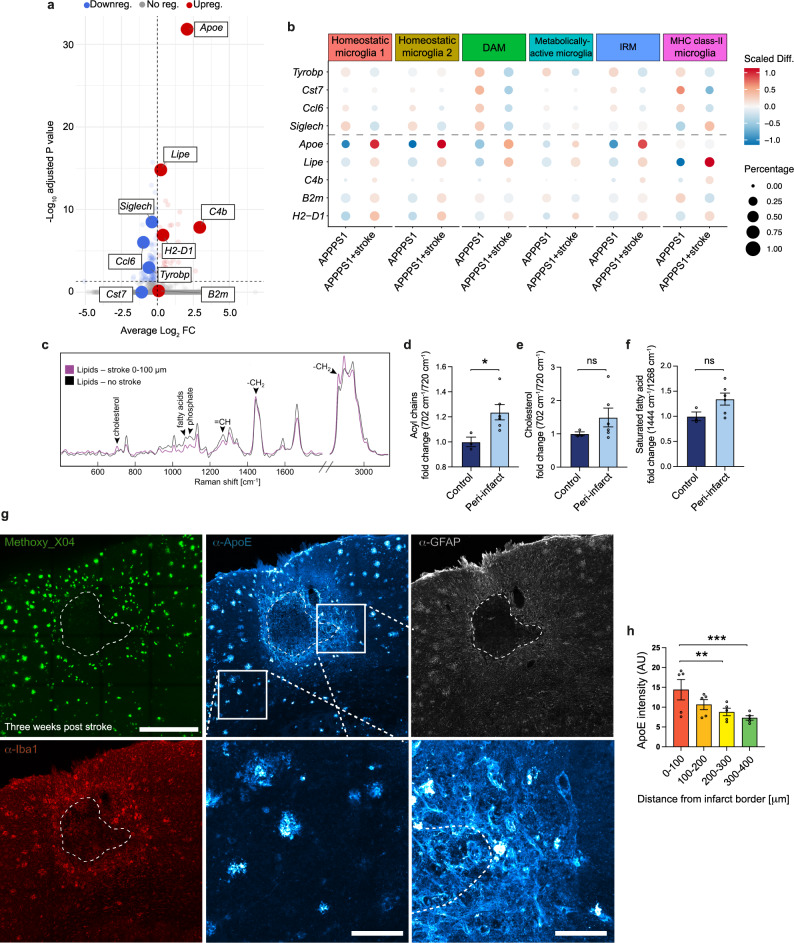
*Apoe* is highly enriched in microglia after ischemic stroke in APPPS1 mice **a**. Volcano plot of pseudobulk analysis of microglia in APPPS1 + stroke vs. APPPS1. **b** Dot plot illustrating gene expression changes in each identified microglial cluster in APPPS1 and APPPS1 stroke. **c** Averaged Raman spectra of peri-infarct Aβ plaques and Aβ plaques from APPPS1 mice without stroke. Markings indicate characteristic peaks of cholesterol, fatty acids and phosphate. **d** Peak ratio analysis of Raman spectra indicating acyl chains **e** cholesterol and **f** saturated fatty acid (*n* = 3 mice three weeks post stroke, two Aβ plaques per Sect. (1 male, 2 females), *n* = 3 mice no stroke APPPS1 controls, one Aβ plaque per Sect. (2 males, 1 female). **g** Representative maximum intensity projection illustrating ApoE immunoreactivity in three weeks post-stroke APPPS1 mice. Scale bar = 500 μm, 100 μm insets. **h** ApoE intensity is significantly higher in close proximity to the infarct core (3 males, 2 females). Two-tailed unpaired t-test **d**, **e**, **f**. Repeated-measures one-way ANOVA with Tukey’s multiple comparison test **h**. * = *p* < 0.05, ** = *p* < 0.01, *** = *p* < 0.001, **** = *p* < 0.0001. For full statistical details, see Supplementary Table 2

To elucidate how ischemic injury alters microglial function in the presence of cerebral-β-amyloidosis we next compared APPPS1 + stroke to APPPS1. To first obtain an integrated overview, we performed a pseudo-bulk cross-cluster differential expression analysis, which identified *Apoe* as the most significantly upregulated gene across the microglial compartment as a whole, accompanied by *C4b*, *H2-D1*, *B2m* and *Lipe* among the top upregulated genes, while *Tyrobp*, *Cst7*, *Ccl6* and *Siglech* were consistently downregulated (Fig. 5a). This pointed toward a shift away from canonical DAM effector function and towards a lipid-metabolic, *Apoe*-dominated program. No significant changes were identified in the metabolically active microglia cluster or interferon-response microglia. In the MHC class II antigen-presenting cluster, six genes were found to be significantly upregulated, however given the very small size of this cluster (especially in comorbidity), these findings were not investigated further. Focusing our attention towards the homeostatic and DAM clusters, remarkably we found that ischemic stroke in APPPS1 mice results in distinct yet convergent transcriptional changes in both homeostatic and DAM populations. We next analyzed transcriptional alterations within each cluster and notably, both homeostatic clusters exhibited upregulation of *Apoe*, *H2-D1* and *C4b* in APPPS1 + stroke relative to APPPS1, suggesting partial transcriptional remodeling of the homeostatic pool towards enhanced lipid handling, complement-mediated clearance and MHC class I antigen presentation under co-morbid conditions (Fig. 5b). Within the homeostatic clusters, *Siglech*, a canonical marker of microglial quiescence, was significantly downregulated in homeostatic microglia 1 in APPPS1 + stroke relative to APPPS1, reinforcing the partial breakdown of the typical homeostatic identity described above (Fig. 5b). Homeostatic microglia 1 additionally exhibited upregulation of *B2m*, a stage 1 DAM-associated gene, while homeostatic microglia 2 showed upregulation of *Lipe*, pointing to divergent transcriptional trajectories within the homeostatic compartment: early DAM-like priming in homeostatic microglia 1 and lipid-metabolic activation in homeostatic microglia 2 (Fig. 5b). Within the DAM cluster, *Apoe* expression was further elevated in APPPS1 + stroke compared to APPPS1, yet the canonical DAM genes *Tyrobp*, *Cst7* and *Ccl6* were simultaneously downregulated - accompanied by upregulation of *Lipe* and continued suppression of *Siglech* (Fig. 5b). The parallel rise of *Apoe* and lipid-metabolic genes alongside the decrease of TREM2-associated effectors was therefore a consistent feature across both homeostatic and DAM cluster compartments. Taken together, these findings indicate that ischemic stroke induces changes in the microglial transcriptional landscape, including a shift away from canonical DAM effector function towards an *Apoe*-dominated, lipid-metabolic program. Critically, this does not represent a simple amplification of the AD-associated DAM response, but a qualitatively distinct remodeling in which lipid handling supersedes lysosomal clearance as the dominant microglial output.

Since multiple distinct microglial clusters converged on upregulation of *Apoe* and remodeling of lipid-metabolic pathways, we employed RAMAN microscopy to assess changes in lipid composition within peri-infarct Aβ plaques compared to controls (Fig. 5c). We identified a significant increase in acyl chain content within peri-infarct Aβ plaques, alongside a trend towards increased cholesterol and saturated fatty acid levels (Fig. 5b–d), indicative of a denser and less fluid lipid environment consistent with augmented Aβ aggregation and compaction in the co-morbid state. To assess ApoE protein distribution directly, we performed ApoE immunohistochemistry in three-week post-stroke APPPS1 mice (Fig. 5g), which revealed an abundance of ApoE immunoreactivity specifically within the peri-infarct region, precisely where augmented Aβ plaque compaction was identified (Fig. 5h). Taken together, these findings suggest that ischemic stroke in combination with cerebral β-amyloidosis drives a shift in microglial phenotype towards enhanced lipid handling and pronounced *Apoe* upregulation, which together may underlie the concurrent increase in Aβ plaque compaction observed in the co-morbid condition.

**Fig. 6:**
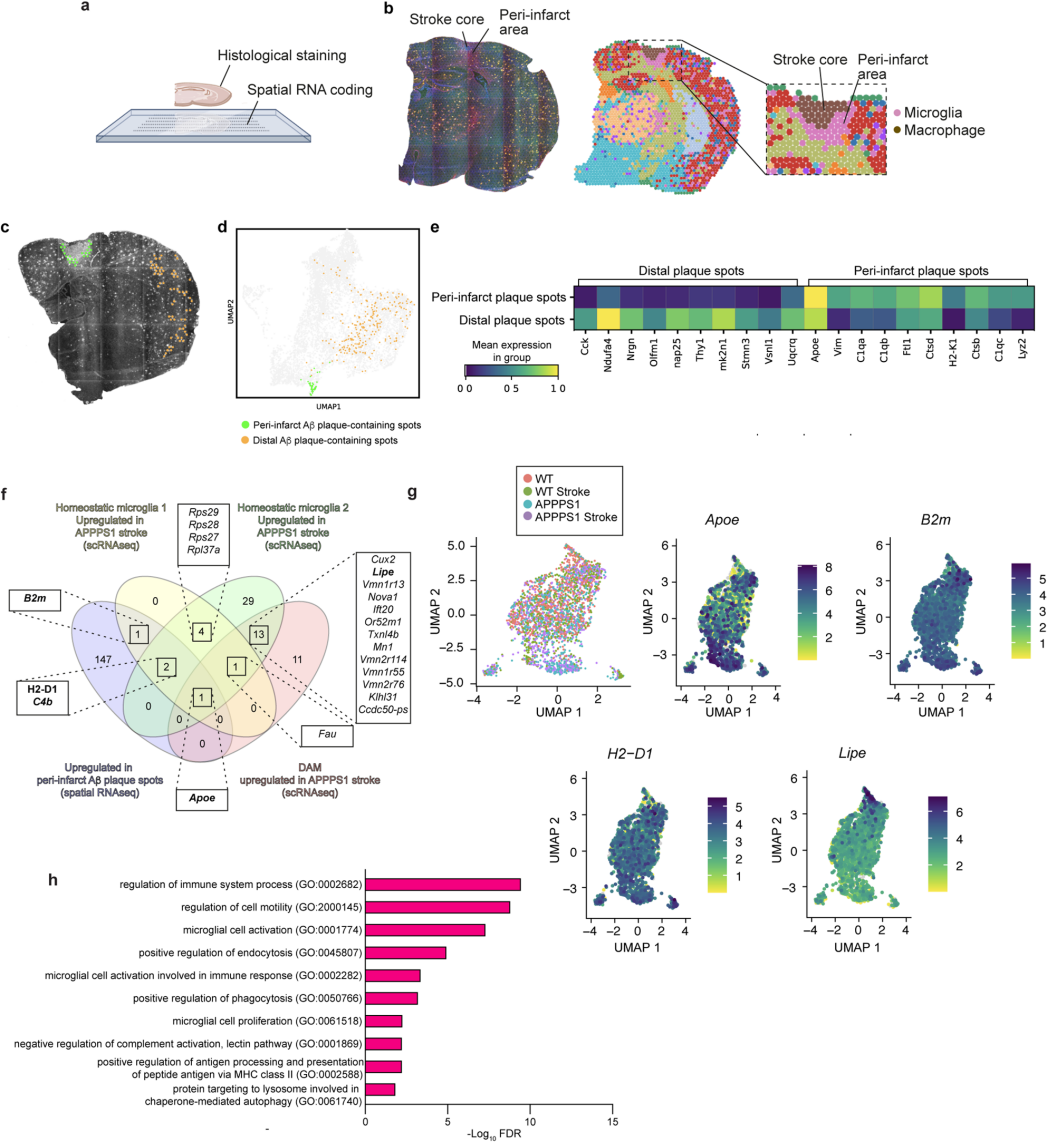
Spatial transcriptomics further consolidate augmented *Apoe* expression as a hallmark of microglia in APPPS1 + stroke. **a** Graphical representation of spatial RNA-seq. **b** Automatic cell detection for microglia and macrophages. Note that macrophages are present within the infarct core whereas microglia are found throughout the brain parenchyma including the peri-infarct area. **c** Immunohistochemical labelling of Aβ plaques (CN6), GFAP and microglia (CD45) on an APPPS1 coronal brain section from Visium spatial transcriptomics of a three weeks post-stroke APPPS1 section with representative images of manually selected peri-infarct Aβ plaques (right, shown in green) and distal Aβ plaques (shown orange) for subsequent transcriptomic analysis. **d** UMAP showing clustering of all spots from *n* = 3 APPPS1 coronal sections three weeks post-stroke (from individual mice) used in spatial transcriptomic experiments. Peri-infarct Aβ plaque-containing spots are shown in green and distal Aβ plaque-containing spots are shown in orange. **e** Heatmap showing differentially expressed genes between peri-infarct Aβ plaque-containing spots and distal Aβ plaque-containing spots. **f** Venn diagram showing the overlap between upregulated genes from homeostatic microglia 1 from APPPS1 stroke mice (scRNA-seq), homeostatic microglia 2 from APPPS1 stroke mice (scRNA-seq), DAM from APPPS1 stroke mice (scRNA-seq) and top upregulated genes in peri-infarct Aβ plaque-containing spots compared to distal Aβ plaque-containing spots. **g** UMAP plots of illustrating the expression levels of *Apoe*, *B2m*, *H2-D1* and *Lipe*. **h** Bar chart illustrating significantly enriched GO-terms in peri-infarct Aβ plaque-containing spots compared to distal Aβ plaque-containing spots. (For spatial transcriptomics, *n* = 3 three weeks post stroke mice, 2 females, 1 male)

Leveraging the precise spatial boundary of the ischemic stroke, we employed spatial transcriptomics (Fig. 6a) on APPPS1 mice three weeks post-stroke. Using this approach we can attain a spatial resolution of ~ 55 μm per spot (therefore not single cell resolution) for subsequent comparison with our scRNAseq approach that lacks spatial resolution, thus partially compensating for the technical limitations of each complimentary approach. Automated cell-type annotation indicated that invading macrophages were confined to the infarct core, while cells in the peri-infarct area were identified as microglia (Fig. 6b), consistent with our previous immunofluorescence data showing TMEM119⁺ microglia in the peri-infarct region (Fig. 3e). Next, we manually identified spots containing peri-infarct Aβ plaques via immunohistochemical labelling of Aβ for comparison to spots containing distal Aβ plaques. Clustering analysis further demonstrated that spots containing microglia associated with distal Aβ plaques (orange) were clearly separable from those containing peri-infarct Aβ plaque-associated microglia (green), the latter forming a tight cluster indicative of highly similar gene expression profiles (Fig. 6c, d). Comparing peri-infarct to distal Aβ plaque-containing spots (Fig. 6e, Extended Data Table 1), we identified several microglia-related genes upregulated in the peri-infarct region, including *Apoe*, complement proteins (*C1qa*, *C1qb*, *C3*) and lysosome-related genes (*Ctsb*, *Ctsd*).

To integrate our spatial and single-cell datasets, we compared genes upregulated in homeostatic microglia 1, homeostatic microglia 2 and DAM from APPPS1 + stroke mice in our scRNA-seq data with genes upregulated in peri-infarct Aβ plaque-containing spots. In this four-set Venn analysis (Fig. 6f), *Apoe* emerged as the sole gene upregulated across all four datasets, identifying it as a conserved core of the peri-infarct microglial response. *B2m*, *H2-D1* and *C4b* were additional shared genes between homeostatic clusters 1 and 2 with peri-infarct Aβ plaque-containing spots. Meanwhile homeostatic microglia 2 and DAM displayed a broader shared gene programme, suggesting partial transcriptional convergence between the homeostatic and DAM states under co-morbid conditions. Taken together, these findings identify a coordinated, cross-cluster microglial response in APPPS1 + stroke characterised by pronounced upregulation of *Apoe*, spatially anchored to the peri-infarct Aβ plaque microenvironment and directly consistent with the *Apoe*-dominated lipid-metabolic shift identified in our bulk and per-cluster differential expression analyses.

Gene ontology analysis of the top upregulated genes in peri-infarct Aβ plaque-containing spots relative to distal spots revealed enrichment across three functional themes: phagocytic and endocytic clearance (positive regulation of phagocytosis and endocytosis), antigen processing and immune priming (positive regulation of antigen processing and presentation via MHC class II), and lysosomal targeting and autophagy (protein targeting to lysosome involved in chaperone-mediated autophagy), alongside microglial cell activation and negative regulation of complement activation via the lectin pathway (Fig. 6h, Extended Data Table 1). Intriguingly, regulation of cell mobility was also enriched in peri-infarct Aβ plaque containing spots, consistent with augmented microglial dynamics to engage with Aβ plaques. Notably, this GO enrichment profile closely mirrors that reported in a study characterizing a resilient microglial phenotype in human Alzheimer’s disease samples, in which microglia associated with dense-core Aβ plaque compaction show enrichment of the same phagocytic, lysosomal and immune-activating pathways [16].

**Fig. 7:**
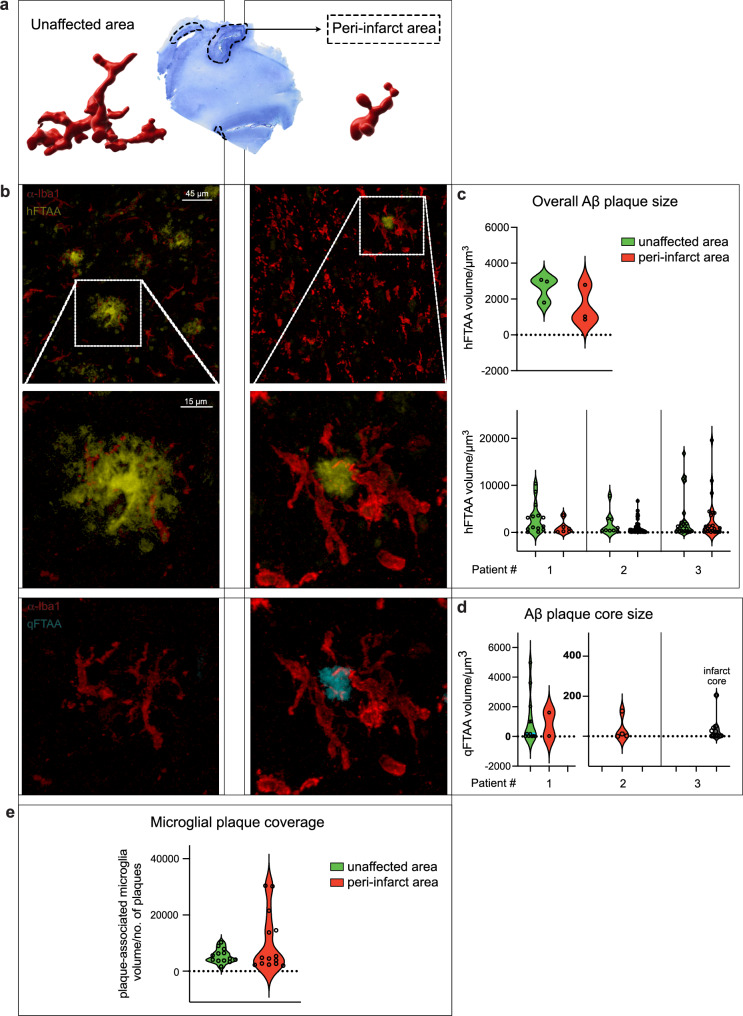
Peri-infarct Aβ plaques in humans are morphologically consistent with peri-infarct Aβ plaques in mice. **a** 3D reconstruction of microglia in human stroke tissue (Nissl stain shown in blue) and in unaffected brain regions. **b** Representative images of hFTAA- and qFTAA-labelled Aβ plaques in human stroke brain tissue. Note the striking morphological difference between Aβ plaques in the peri-infarct region compared to unaffected brain tissue. **c** Violin plot illustrating hFTAA volume of Aβ plaques in peri-infarct and unaffected tissue (top) with individual patient data shown below. **d** Violin plot illustrating qFTAA volume of Aβ plaque cores in individual patients. **e** Violin plot illustrating microglial Aβ plaque coverage in peri-infarct and unaffected brain tissue

In order to address the clinical relevance of our finding, we analyzed human brain sections to determine whether augmented Aβ plaque compaction also occurs in humans post-stroke. We examined brain samples from three patients with ischemic stroke who also exhibited Aβ deposition. Despite the small sample number and an unclear timeline of the disease events, we found that human peri-infarct Aβ plaques were morphologically similar to the peri-infarct Aβ plaques identified in our mouse model (Fig. 7a-b). We observed a trend towards reduced hFTAA volume in the peri-infarct region (Fig. 7c), consistent with our findings in the mouse model. Correspondingly, two out of three stroke patient samples showed a trend towards increased qFTAA volume in the peri-infarct region (Fig. 7b, d, e), suggesting a higher rate of Aβ plaque compaction within the peri-infarct area. Analysis of microglial engagement further revealed a trend towards increased microglial Aβ plaque coverage in the peri-infarct region. Collectively, these human data support the hypothesis that the mechanisms uncovered in this study regarding improvement in microglia-mediated Aβ plaque compaction in mouse models may also occur in humans.

Taken together, these data demonstrate that the pathological milieu arising from the combination of ischemic stroke and cerebral β-amyloidosis enhances microglial activity in a manner that promotes the formation of comparatively inert, compacted Aβ plaques and is associated with reduced small fibrillar deposits of immature Aβ and decreased axonal dystrophy in the peri-infarct region. This phenotype is reminiscent of the microglial and Aβ plaque characteristics reported in resilient AD patients, suggesting that stroke-induced neuroinflammation may paradoxically engage a neuroprotective microglial program relevant to human disease resilience.

## Discussion

Modulation of microglial function is becoming an increasingly appealing target for disease-modifying treatment of neurodegenerative diseases [32]. Microglia can directly contribute to neuroinflammation in diverse conditions such as AD, Parkinson’s disease, multiple sclerosis, frontotemporal dementia, traumatic brain injury and ischemic stroke. Therefore, abrogation of neuroinflammation holds potential as a therapeutic strategy to prevent further neurological damage. Additionally, however, microglia can also be beneficial in many of these conditions (such as via phagocytosis of cellular debris and protein aggregates such as Aβ in AD and α-synuclein in Parkinson’s disease for example). It is prudent therefore to investigate how microglia respond not only in each individual pathology but also in co-morbidity, since microglial function may be altered in the co-morbid state compared to a single pathology. Furthermore, many elderly patients in which these neurological diseases occur present with multiple comorbidities. Therefore, it is important to attain insight into the pathophysiological behavior of these cells both spatially – and temporally – as microglial phenotypes and cellular responses can vary not only between conditions but also over disease progression. By elucidating these idiosyncratic microglial phenotypes, we can develop a better comprehension of how we may be able to pharmacologically facilitate beneficial microglial phenotypes and to do this at the appropriate time in the course of each disease.

Investigating the effects of an ischemic insult, we here demonstrate that the somewhat dormant homeostatic microglial pool in cerebral β-amyloidosis model (and despite the abundance of DAM present in these mice), are able to partially regain beneficial functionality that allows them to phagocytically remove small fibrillar deposits and to compact large Aβ plaques; provided that they receive the appropriate external stimulus. While this study did not focus on age or sex differences, we did not observe any significant differences in this regard (Extended Data Fig. 9). It is important to note that the brain damage caused by ischemic injury certainly outweighs any potential benefit that could be attained from improved microglial function; however, this finding opens the possibility that with the right therapeutic approach, such beneficial microglial functions may be triggered in AD patients. Additionally, since the APPPS1 mouse model exhibits only mild cognitive impairment, a focal cortical stroke would be expected to dominate global behavior and mask any potential cognitive benefit resulting from augmented Aβ plaque compaction and reduced Aβ plaque-associated axonal dystrophy in the peri-infarct area. Future work therefore must aim to pharmacologically promote this phenotypic alteration in microglial function in the absence of cerebral ischemia.

Within the infarct core we saw a nearly complete reduction in Aβ plaque load. Our data indicate that this phenomenon is mediated by macrophages confined to the infarct core. Thus, our focus remained on the accumulation of Aβ plaques in the peri-infarct region driven by brain-resident microglia. Strikingly, in addition to augmented dense-core Aβ plaque formation, we observed a stark loss of non-Aβ plaque associated deposits of immature/prefibrillar Aβ in the peri-infarct region. In APPPS1 mice (that develop Aβ plaques already at six weeks of age), new dense core Aβ plaque production is abrogated after five months of age followed by an accumulation of immature Aβ species in the brain parenchyma. Yet, despite this cessation of microglial function in terms of Aβ plaque compaction, the pathological milieu resulting from the co-morbid state of ischemic stroke with cerebral β-amyloidosis is sufficient to transform the remaining homeostatic microglia into a state of regained functionality, enabling them to create relatively benign dense-core Aβ plaques.

While the DAM state is initially useful to limit AD pathology, ultimately it is likely not beneficial as pro-inflammatory signaling gains the upper hand [12]. This often has led to the characterization of the microglial response as a double-edged sword. Our data challenge the notion that this is an inevitable, fixed trajectory. Rather than simply amplifying canonical DAM, ischemic injury in the context of cerebral β-amyloidosis engaged a distinct transcriptional state not seen in response to Aβ pathology alone, demonstrating that the microglial compartment retains a degree of plasticity that can be unlocked by a secondary stimulus. This is conceptually important: it implies that the transition toward a detrimental pro-inflammatory phenotype is not predetermined, and that under appropriate conditions, microglia can mount a qualitatively different - and in this context functionally superior - response to Aβ. The precise upstream signals driving this reprogramming remain to be established, but the peri-infarct microenvironment is rich in damage-associated molecular patterns and inflammatory mediators that could prime microglia toward altered lipid handling, potentially through nuclear receptor pathways such as PPAR-γ or LXR, both of which are known regulators of *Apoe* expression and lipid metabolism in myeloid cells [33, 34].

When comparing APPPS1 + stroke to APPPS1 within individual clusters, both homeostatic and DAM populations underwent distinct yet convergent transcriptional alterations. In both homeostatic clusters, stroke induced increased expression of *Apoe*, *H2-D1*, and *C4b* alongside reduced *Siglech*, indicating a shift away from classical homeostasis toward a state associated with altered lipid handling, complement engagement, and immunological priming. The upregulation of complement components *C4b* and *H2-D1* is particularly noteworthy: complement-mediated opsonisation of Aβ deposits is an established route for microglial phagocytic engagement [35], and increased complement expression in peri-infarct microglia may facilitate plaque coverage and compaction. Within the DAM cluster, *Apoe* was further upregulated, yet accompanied by reduced expression of canonical DAM genes including *Tyrobp*, *Cst7*, and *Ccl6*. This pattern warrants careful consideration: *Tyrobp* encodes DAP12, the signaling adaptor for TREM2, which canonically drives *Apoe* expression during the DAM transition [36]. The simultaneous downregulation of *Tyrobp* and upregulation of *Apoe* therefore suggests that in the comorbid context, *Apoe* expression is driven through an alternative, TREM2-independent pathway in which PPAR-γ and LXR are possible candidates, as both are activated by lipid ligands enriched [33] in the post-ischemic environment and are established transcriptional drivers of *Apo*e in myeloid cells [34]. The beneficial microglial phenotype detected in the peri-infarct region therefore does not reflect a simple amplification of the canonical DAM program, but rather a distinct transcriptional reprogramming. Particularly striking was the consistent upregulation of *Apoe* across both homeostatic and DAM populations, suggesting a common shift toward a lipid-associated state that may underlie the augmented Aβ plaque compaction observed in vivo.

These scRNA-seq findings were reinforced by results from spatial transcriptomics. Peri-infarct Aβ plaque-associated spots formed a transcriptionally distinct and tightly clustered population relative to spots associated with distal Aβ plaques, indicating that the peri-infarct niche imposes a highly stereotyped transcriptional program. Genes upregulated in peri-infarct spots included *Apoe*, complement components, and lysosomal genes - closely mirroring the pathways identified in our scRNA-seq dataset. Most strikingly, cross-dataset integration identified *Apoe* as the sole gene consistently upregulated across peri-infarct Aβ plaque-associated spots and all relevant APPPS1 + stroke microglial clusters. Convergent evidence at the protein level further supported this conclusion: a remarkable accumulation of ApoE in the peri-infarct area was associated with significantly higher acyl group content within Aβ plaques, pointing toward stroke-driven remodeling of lipid handling within the plaque microenvironment. These data demonstrate that ischemic stroke does not simply intensify the classical DAM response to cerebral β-amyloidosis, but instead induces a distinct ApoE-enriched microglial state characterized by altered lipid handling, complement activity, and lysosomal processing. We propose that this transcriptional shift underlies the regained functionality of peri-infarct microglia, enabling them to clear immature Aβ species and compact Aβ plaques into a more inert form. Mechanistically, highly compact Aβ plaques are thought to have reduced surface permeability and release fewer soluble oligomeric Aβ species [37], which may contribute to the reduced axonal dystrophy observed in the peri-infarct region.

The translational significance of these findings is underscored by our analysis of human brain tissue from stroke patients with concomitant Aβ deposition. Given the inherent variability in disease timelines across patients, the observed trends are particularly noteworthy: increased microglial coverage of peri-infarct Aβ deposits, reduced hFTAA labelling, and increased qFTAA labelling in the peri-infarct region collectively suggest an accelerated compaction rate consistent with our mouse model findings. These observations align with a documented enrichment of highly compact Aβ plaques in cognitively resilient AD patients [16], raising the possibility that compact Aβ plaque formation may be associated with resilience to Aβ toxicity. Of particular relevance here is the APOE4 allele, the strongest genetic risk factor for late-onset AD. APOE4 is known to impair microglial lipid handling [38] and reduce ApoE-mediated Aβ clearance [39], raising the question of whether APOE4 carriers may be specifically impaired in their capacity to mount the protective ApoE-enriched microglial response we describe - a deficit that could in part explain the heightened AD risk conferred by this allele. Conversely, this suggests that pharmacological strategies aimed at restoring ApoE-driven lipid handling, or directly engaging the complement and lysosomal pathways we identify, could recapitulate the beneficial microglial phenotype and attenuate Aβ toxicity independently of an ischemic trigger.

## Supplementary Information

Supplementary Material 1. Extended Data Figure 1: Increased Aβ plaque load in APP23 mice three weeks post stroke. (**a**) Experimental strategy to model ischemic stroke in an alternative mouse model of cerebral amyloidosis (APP23 mice). Due to the substantially slower rate of Aβ plaque formation in this model, strokes were induced between 16 months and 22 months of age. (**b**) Representative image of an APP23 mouse brain section three weeks post stroke. Scale bar = 200 μm. (**c**) Significantly higher dense-core Aβ plaque load as well as (**d**) the number of dense core Aβ plaques were found in close proximity to the infarct border three weeks post-stroke in APP23 mice (*n* = 5 mice, 3 males, 2 females). Repeated-measures one-way ANOVA with Tukey’s multiple comparison test. * = *p* < 0.05, ** = *p* <0.01, *** = *p* < 0.001, **** = *p* <0.0001. For full statistical details, see Supplementary Table 2. Extended Data Figure 2: Temporal alterations to Aβ deposits and dystrophic neurite occurrence. (**a-i**) Representative image of an APPPS1 mouse brain section at five, nine and 16 months old. Aβ deposits are labelled with Methoxy X04 (green) and hFTAA (red). (**j**) No significant increase in dense core (Methoxy X04+) Aβ plaque load or the (**k**) number of dense core Aβ plaques occurs after five months of age. However, (**l**) there is a significant increase in the hFTAA covered area (immature Aβ load), indicating an increase in immature Aβ species in the brain parenchyma (n = 3-5 mice: 5 months old 2 males, 1 female, 9 months old: 3 males, 2 females, 16 months old: 3 males 2 females). (m) False-color coding of spectral scan images of APPPS1 brain sections stained with hFTAA and qFTAA taken at five, nine and 16 months old. Note the accumulation of immature (red shifted) Aβ deposits in the brain parenchyma (**n-p**). No significant difference in Aβ-plaque associated axonal dystrophy was present in close proximity to the infarct border (n = 4 mice, 2 males, 2 females) at one week post stroke, however (**q-s**) significantly less Aβ plaque associated axonal dystrophy was present in close proximity to the infarct border (n = 7 mice, 3 males, 4 females) at nine weeks post stroke. Dense-core Aβ plaques (n,o,q,r) are visible in green (labelled with Methoxy_X04), glial scar is visible in white (GFAP immunoreactivity), dystrophic neurites are visible in red (amyloid precursor protein (APP) immunoreactivity). (**j**, **k**, **l**) Ordinary one-way ANOVA with Tukey’s multiple comparison test. (**p**, **s**) Repeated-measures one-way ANOVA with Tukey’s multiple comparison test. * = *p* < 0.05, ** = *p* <0.01, *** = *p* < 0.001, **** = *p* <0.0001. For full statistical details, see Supplementary Table 2. Extended Data Figure 3: LAMP1 immunoreactivity is decreased proximal to the infarct core. (**a**) Representative image of LAMP1 immunoreactivity surrounding peri-infarct Aβ plaques. Scale bar = 50 μm. (**b**) LAMP1 immunoreactivity surrounding Aβ plaques was significantly higher distal to the infarct core (n = 5 mice, 2 males, 3 females). (**c**) Representative images of contralateral hFTAA, Methoxy_X04 and neurotrace at three weeks post stroke. (**d**) Isolated extraneuronal and (**e**) intraneuronal hFTAA and corresponding surface reconstruction. Scale bar = 80 μm. (**f**) Quantification of intraneuronal and extraneuronal hFTAA (n = 3 mice, 1 male, 2 females). Repeated-measures one-way ANOVA with Tukey’s multiple comparison test. * = *p* < 0.05, ** = *p* <0.01, *** = *p* < 0.001, **** = *p* <0.0001. For full statistical details, see Supplementary Table 2. Extended Data Figure 4: Alterations upon microglia depletion with pexidartinib. (**a**) Representative image of an APPPS1 mouse brain section at three weeks post stroke. Aβ deposits are labelled with Methoxy X04 (green) and microglia are labelled with anti-Iba1 (orange). Scale bar = 50 μm, inset 20 μm. (**b**) Iba1-immunoreactive area around Aβ plaques is significantly higher proximal to the infarct border (n = 5 mice, 3 males, 2 females). (**c**, left) Representative image of an APPPS1 mouse brain section at three weeks post stroke. Aβ deposits are labelled with Methoxy X04 (green), glial scar is visible in white (anti-GFAP, white) and microglial nuclei are labelled with anti-PU.1 (red). Scale bar = 20 μm. (**c**, right) Representative image of segmentation procedure used to quantify microglial nuclei around peri-infarct Aβ plaques. (**d**) The number of proximal PU.1+ nuclei per μm3 of Aβ plaque volume is significantly higher proximal to the infarct border (n = 6 mice, 4 males, 2 females). (**e**) Representative images of anti-Iba1 (red) and Methoxy_X04 (green) labelling in the peri-infarct region and contralateral hemisphere three weeks post stroke with or without pexidartinib treatment. Note the reduction of non-Aβ plaque-associated Iba1 immunoreactivity in pexidartinib treated mice compared to standard diet. (**f**) Pexidartinib treatment resulted in a significant reduction in Iba1+ area in the peri-infarct region (n = 5 standard diet mice, 3 males, 2 females, n = 5 pexidartinib-treated mice, 3 males, 2 females) and (**g**) a significant reduction in the qFTAA/hFTAA spectral ratio in the peri-infarct region compared to standard diet three weeks post-stroke (n = 5 standard diet mice, 3 males, 2 females, n = 11 pexidartinib-treated mice, 7 males, 4 females). (**h**) Representative image of an APPPS1 mouse brain section at three weeks post stroke after six weeks of pexidartinib treatment. Aβ deposits are labelled with Methoxy X04 (green), the glial scar is visible in white (anti-GFAP) and dystrophic neurites (anti-APP) are shown in red. Scale bar = 200 μm. (**b**, **d**) Repeated-measures one-way ANOVA with Tukey’s multiple comparison test. (**f**, **g**) Ordinary one-way ANOVA with Šidák’s multiple comparison test (f,g).* = *p* < 0.05, ** = *p* <0.01, *** = *p* < 0.001, **** = *p* <0.0001. For full statistical details, see Supplementary Table 2. Extended Data Figure 5: Neurofilament M-occupied area is not significantly different in the presence of pexidartinib. (**a**) Representative images of neurofilament M immunoreactivity in the peri-infarct region three weeks post stroke from mice either on standard diet or (**b**) pexidartinib (n = 5 standard diet mice, 3 males 2, females, n = 5 pexidartinib-treated mice, 3 males, 2 females). (**c**) No significant difference was found in the area covered by neurofilament M immunoreactivity between mice treated with pexidartinib or standard diet. Scale bar = 200 μm and 100 μm for insets. Ordinary one-way ANOVA with Holm-Šidák’s multiple comparison test (**c**).* = *p* < 0.05, ** = *p* <0.01, *** = *p* < 0.001, **** = *p* <0.0001. For full statistical details, see Supplementary Table 2. Extended Data Figure 6: Validation of microglial sorting strategy. (**a**) Representative snapshots illustrating the gating strategy utilized to FACS-sort microglia from (**b**) wildtype, wildtype stroke, APPPS1 and APPPS1 stroke mice. (**c**) UMAP illustrating the results of automatic cell type detection of FACS-sorted microglia prior and (**d**) after exclusion of non-microglial cells. (**e-j**) Ridge plots illustrating the expression level of microglia marker genes within each microglial cluster. Extended Data Figure 7: Gene expression profiles of known microglial subtypes. Ridge plots illustrating the expression of marker genes of previously published microglial subtypes. Extended Data Figure 8: Marker genes determining different pseudotime trajectories. (**a**) Marker genes (upregulated left column, downregulated right column) for the pseudotime trajectories from homeostatic microglia 1 to metabolically-active microglia, (**b**) homeostatic microglia 1 to MHC-class II microglia and (**c**) homeostatic microglia 1 to IRM. Extended Data Figure 9: No sex or age differences were observed in the study. (**a**) Inter-age comparison of dense core Aβ plaque load at three weeks post stroke suggests no age dependent phenotype. Note that only groups with n = 3 or higher were used for statistical analysis. (**b**) Similarly, no differences were observed in dense core Aβ plaque load, (**c**) the radio of APP to Aβ plaque area or (**d**) the dense core Aβ plaque load after pexidartinib treatment between males and females. Ordinary one-way ANOVA with Šidák’s multiple comparison test. * = *p* < 0.05, ** = *p* <0.01, *** = *p* < 0.001, **** = *p* <0.0001. For full statistical details, see Supplementary Table 2.

Supplementary Material 2. Extended Data Video 1: One week post stroke iDISCO-cleared mouse brain. Video showing hFTAA labelling in an iDISCO-cleared one week post stroke mouse brain.

Supplementary Material 3. Extended Data Video 2: Three weeks post stroke iDISCO-cleared mouse brain. Video showing hFTAA labelling in an iDISCO-cleared three weeks post stroke mouse brain.

Supplementary Material 4. Supplementary Table 1: Antibodies. Supplementary table detailing which antibodies were used in the study.

Supplementary Material 5. Supplementary Table 2: Statistics. Supplementary table detailing statistical analyses used in the study.

Supplementary Material 6. Supplementary Table 3: Human samples. Supplementary table providing details regarding human brain sections used in the study.

Supplementary Material 7. Extended Data Table 1: DEGs and GO term analyses

Supplementary Material 8. Methods

## Data Availability

Any additional requests for data should be addressed to Prof. Jasmin Hefendehl (hefendehl@bio.uni-frankfurt.de). Details regarding scRNAseq analysis and data can be found at the following websites respectively. https://github.com/KJPMolgenLab/scSeq_Hefendehl/ and https://kjpmolgenlab.github.io/scSeq_Hefendehl/.

## Code availability

The code for segmentation of qFTAA- and hFTAA- labelled Aβ plaques can be found here: https://gitlab.mpcdf.mpg.de/mpibr/scic/roioverstack. Any additional requests for code should be addressed to Prof. Jasmin Hefendehl (hefendehl@bio.uni-frankfurt.de).
