# Supplementary material for: Ischemic injury triggers a protective microglial phenotype in models of Aβ pathology
Source: J Neuroinflammation. 2026 Jun 9;23:213. doi: 10.1186/s12974-026-03897-x (PMC13292430; doi:10.1186/s12974-026-03897-x)
Supplement: Supplementary file 1 — Supplementary Material 1. Extended Data Figure 1: Increased Aβ plaque load in APP23 mice three weeks post stroke. (a) Experimental strategy to model ischemic stroke in an alternative mouse model of cerebral amyloidosis (APP23 mice). Due to the substantially slower rate of Aβ plaque formation in this model, strokes were induced between 16 months and 22 months of age. (b) Representative image of an APP23 mouse brain section three weeks post stroke. Scale bar = 200 μm. (c) Significantly higher dense-core Aβ plaque load as well as (d) the number of dense core Aβ plaques were found in close proximity to the infarct border three weeks post-stroke in APP23 mice (n = 5 mice, 3 males, 2 females). Repeated-measures one-way ANOVA with Tukey’s multiple comparison test. * = p < 0.05, ** = p <0.01, *** = p < 0.001, **** = p <0.0001. For full statistical details, see Supplementary Table 2. Extended Data Figure 2: Temporal alterations to Aβ deposits and dystrophic neurite occurrence. (a-i) Representative image of an APPPS1 mouse brain section at five, nine and 16 months old. Aβ deposits are labelled with Methoxy X04 (green) and hFTAA (red). (j) No significant increase in dense core (Methoxy X04+) Aβ plaque load or the (k) number of dense core Aβ plaques occurs after five months of age. However, (l) there is a significant increase in the hFTAA covered area (immature Aβ load), indicating an increase in immature Aβ species in the brain parenchyma (n = 3-5 mice: 5 months old 2 males, 1 female, 9 months old: 3 males, 2 females, 16 months old: 3 males 2 females). (m) False-color coding of spectral scan images of APPPS1 brain sections stained with hFTAA and qFTAA taken at five, nine and 16 months old. Note the accumulation of immature (red shifted) Aβ deposits in the brain parenchyma (n-p). No significant difference in Aβ-plaque associated axonal dystrophy was present in close proximity to the infarct border (n = 4 mice, 2 males, 2 females) at one week post stroke, however (q-s) significa [file 12974_2026_3897_MOESM1_ESM.docx]

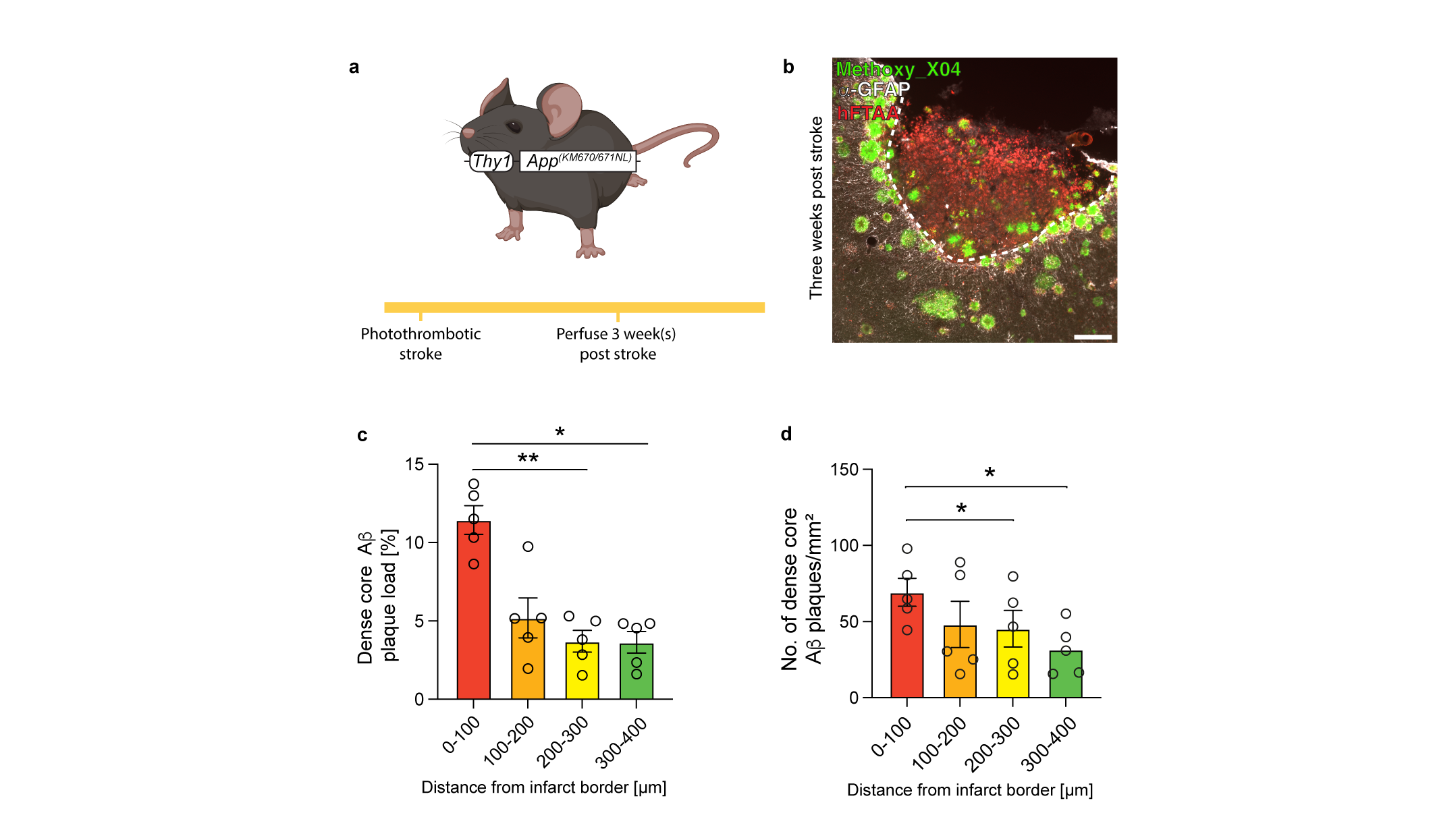


**Extended Data Figure 1.** **Increased Aβ plaque load in APP23 mice three weeks post stroke.** (**a**) Experimental strategy to model ischemic stroke in an alternative mouse model of cerebral amyloidosis (APP23 mice). Due to the substantially slower rate of Aβ plaque formation in this model, strokes were induced between 16 months and 22 months of age. (**b**) Representative image of an APP23 mouse brain section three weeks post stroke. Scale bar = 200 μm. (**c**) Significantly higher dense-core Aβ plaque load as well as (**d**) the number of dense core Aβ plaques were found in close proximity to the infarct border three weeks post-stroke in APP23 mice (n = 5 mice, 3 males 2 females). Repeated-measures one-way ANOVA with Tukey’s multiple comparison test. * = p < 0.05, ** = p <0.01, *** = p < 0.001, **** = p <0.0001. For full statistical details, see Supplementary Table 2.


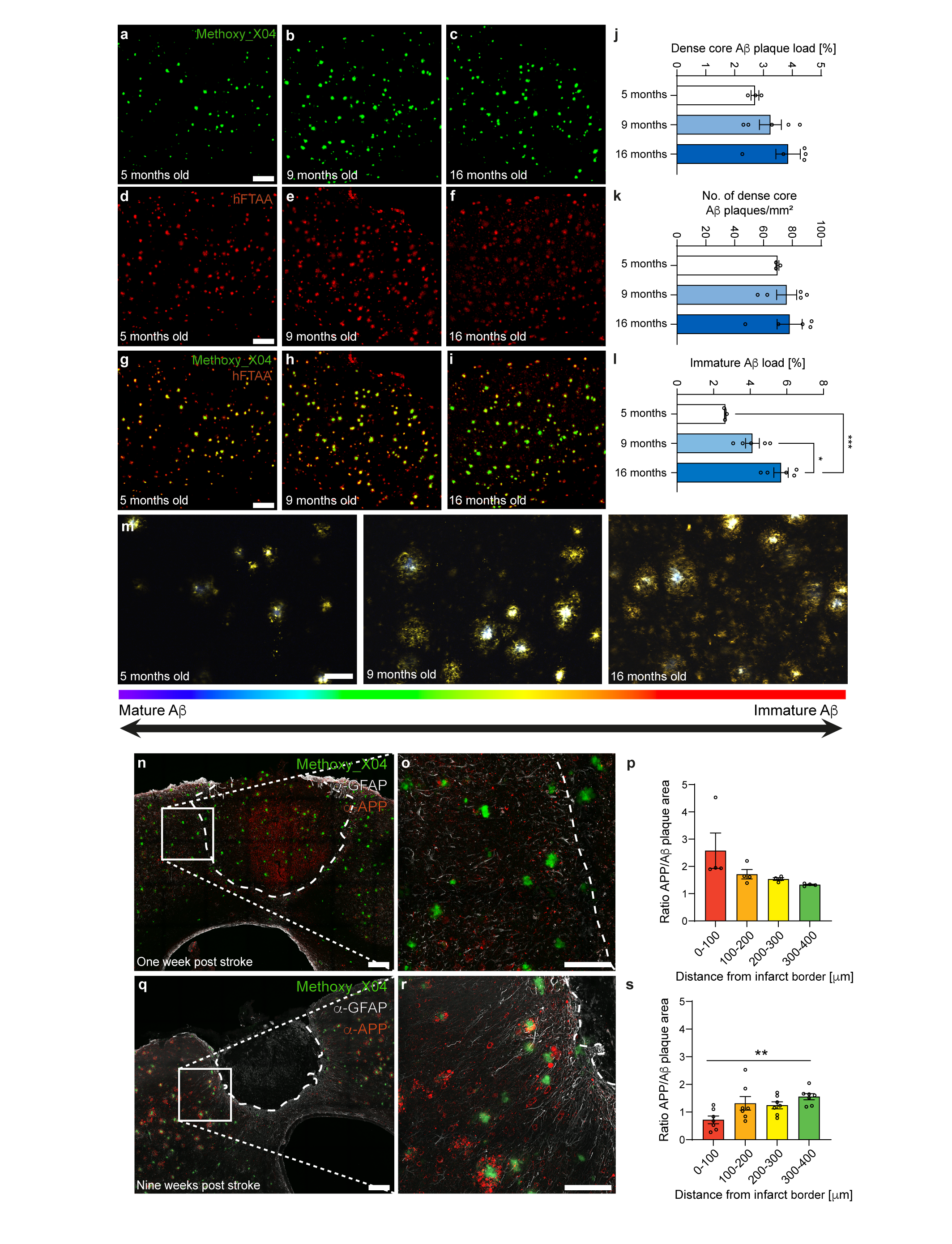


**Extended Data Figure 2. Temporal alterations to Aβ deposits and dystrophic neurite occurrence.** (**a-i**) Representative image of an APPPS1 mouse brain section at five, nine and 16 months old. Aβ deposits are labelled with Methoxy X04 (green) and hFTAA (red). (**j**) No significant increase in dense core (Methoxy X04^+^) Aβ plaque load or the (**k**) number of dense core Aβ plaques occurs after five months of age. However, (**l**) there is a significant increase in the hFTAA covered area (immature Aβ load), indicating an increase in immature Aβ species in the brain parenchyma (n = 3-5 mice: 5 months old 2 males 1 female, 9 months old 3 males 2 females, 16 months old 3 males 2 females). (**m**) False-color coding of spectral scan images of APPPS1 brain sections stained with hFTAA and qFTAA taken at five, nine and 16 months old. Note the accumulation of immature (red shifted) Aβ deposits in the brain parenchyma. (**n-p**). No significant difference in Aβ-plaque associated axonal dystrophy was present in close proximity to the infarct border (n = 4 mice, 2 males 2 females) at one week post stroke, however (**q-s**) significantly less Aβ plaque associated axonal dystrophy was present in close proximity to the infarct border (n = 7 mice, 3 males 4 females) at nine weeks post stroke. Dense-core Aβ plaques (n,o,q,r) are visible in green (labelled with Methoxy_X04), glial scar is visible in white (GFAP immunoreactivity), dystrophic neurites are visible in red (amyloid precursor protein (APP) immunoreactivity). (j,k,l) Ordinary one-way ANOVA with Tukey’s multiple comparison test. (p,s) Repeated-measures one-way ANOVA with Tukey’s multiple comparison test. * = p < 0.05, ** = p <0.01, *** = p < 0.001, **** = p <0.0001. For full statistical details, see Supplementary Table 2.


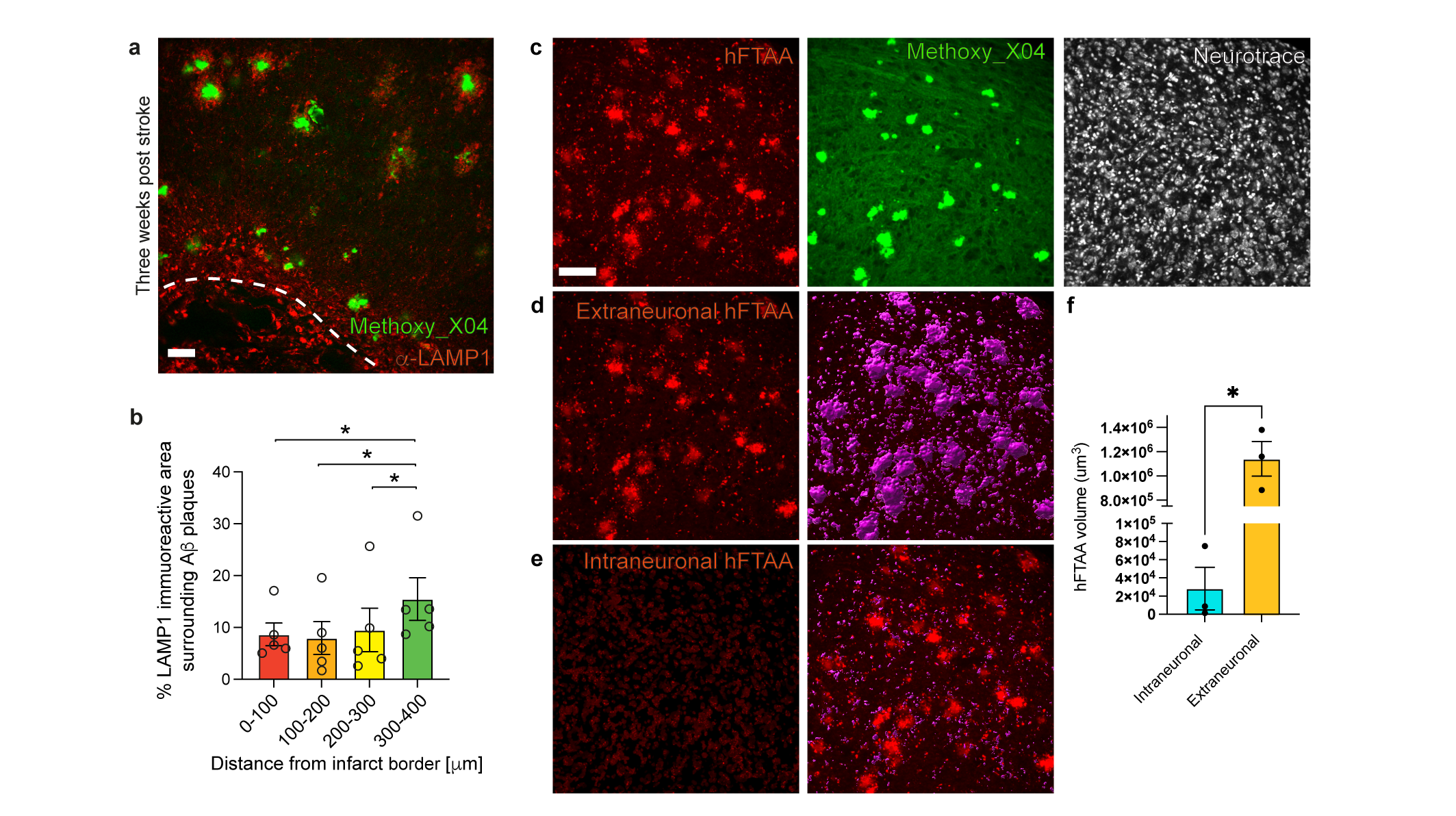


**Extended Data Figure 3.** **LAMP1 immunoreactivity is decreased proximal to the infarct core.** (a) Representative image of LAMP1 immunoreactivity surrounding peri-infarct Aβ plaques. Scale bar = 50 μm. (b) LAMP1 immunoreactivity surrounding Aβ plaques was significantly higher distal to the infarct core (n = 5 mice, 2 males 3 females). (c) Representative images of contralateral hFTAA, Methoxy_X04 and neurotrace at three weeks post stroke. (d) Isolated extraneuronal and (e) intraneuronal hFTAA and corresponding surface reconstruction. Scale bar = 80 μm. (f) Quantification of intraneuronal and extraneuronal hFTAA (n = 3 mice, 1 males 2 females). Repeated-measures one-way ANOVA with Tukey’s multiple comparison test. * = p < 0.05, ** = p <0.01, *** = p < 0.001, **** = p <0.0001. For full statistical details, see Supplementary Table 2.


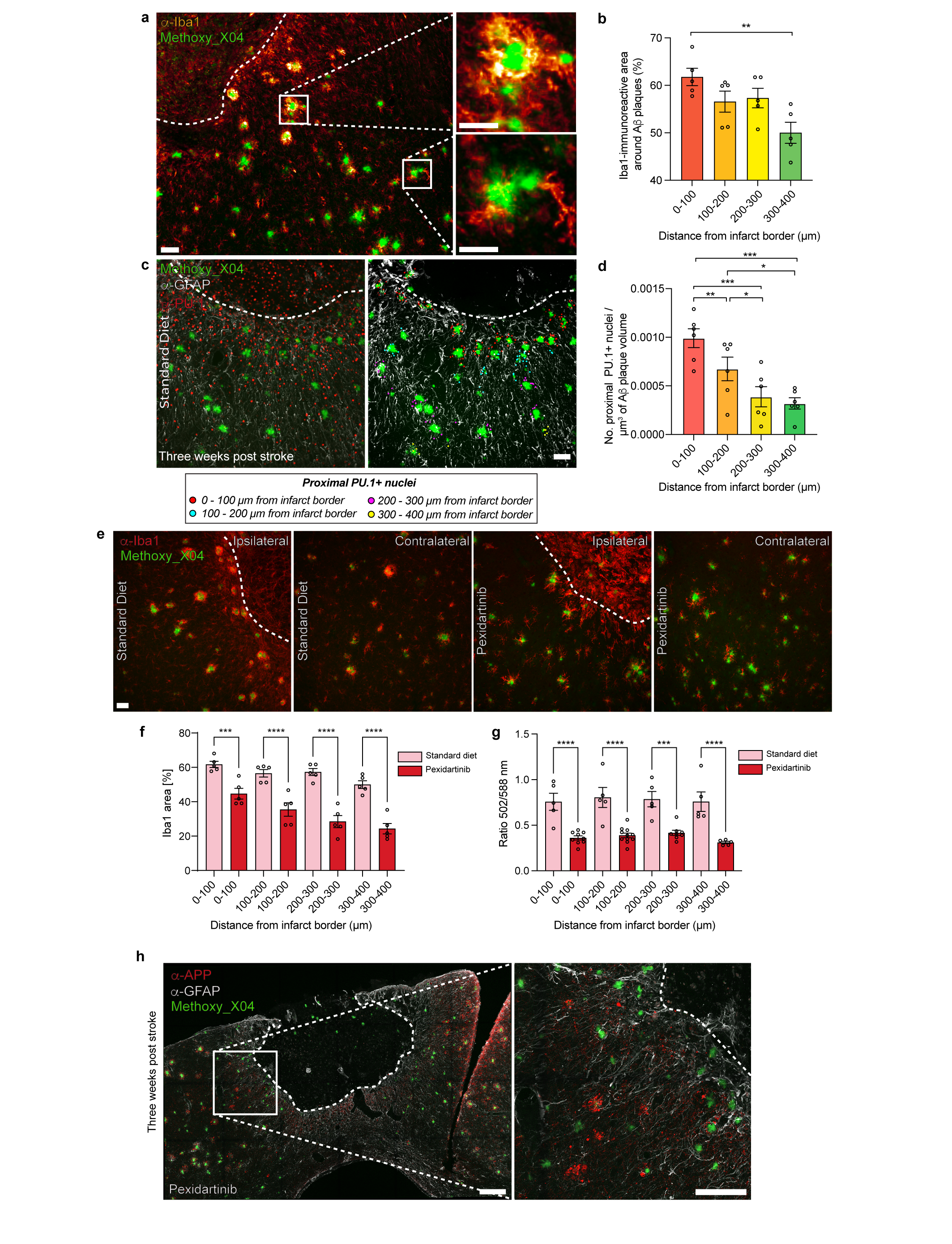


**Extended Data Figure 4. Alterations upon microglia depletion with pexidartinib.** (**a**) Representative image of an APPPS1 mouse brain section at three weeks post stroke. Aβ deposits are labelled with Methoxy X04 (green) and microglia are labelled with anti-Iba1 (orange). Scale bar = 50 μm, inset 20 μm. (**b**) Iba1-immunoreactive area around Aβ plaques is significantly higher proximal to the infarct border (n = 5 mice, 3 males 2 females). (**c**, left) Representative image of an APPPS1 mouse brain section at three weeks post stroke. Aβ deposits are labelled with Methoxy X04 (green), glial scar is visible in white (anti-GFAP, white) and microglial nuclei are labelled with anti-PU.1 (red). Scale bar = 20 μm. (**c**, right) Representative image of segmentation procedure used to quantify microglial nuclei around peri-infarct Aβ plaques. (**d**) The number of proximal PU.1^+^ nuclei per μm^3^ of Aβ plaque volume is significantly higher proximal to the infarct border (n = 6 mice, 4 males 2 females). (**e**) Representative images of anti-Iba1 (red) and Methoxy_X04 (green) labelling in the peri-infarct region and contralateral hemisphere three weeks post stroke with or without pexidartinib treatment. Note the reduction of non-Aβ plaque-associated Iba1 immunoreactivity in pexidartinib treated mice compared to standard diet. (**f**) Pexidartinib treatment resulted in a significant reduction in Iba1^+^ area in the peri-infarct region (n = 5 standard diet mice, 3 males 2 females, n = 5 pexidartinib-treated mice, 3 males 2 females) and (**g**) a significant reduction in the qFTAA/hFTAA spectral ratio in the peri-infarct region compared to standard diet three weeks post-stroke (n = 5 standard diet mice, 3 males 2 females, n = 11 pexidartinib-treated mice, 7 males 4 females). (**h**) Representative image of an APPPS1 mouse brain section at three weeks post stroke after six weeks of pexidartinib treatment. Aβ deposits are labelled with Methoxy X04 (green), the glial scar is visible in white (anti-GFAP) and dystrophic neurites (anti-APP) are shown in red. Scale bar = 200 μm. (b,d) Repeated-measures one-way ANOVA with Tukey’s multiple comparison test. (f,g) Ordinary one-way ANOVA with Šidák’s multiple comparison test (f,g).* = p < 0.05, ** = p <0.01, *** = p < 0.001, **** = p <0.0001. For full statistical details, see Supplementary Table 2.


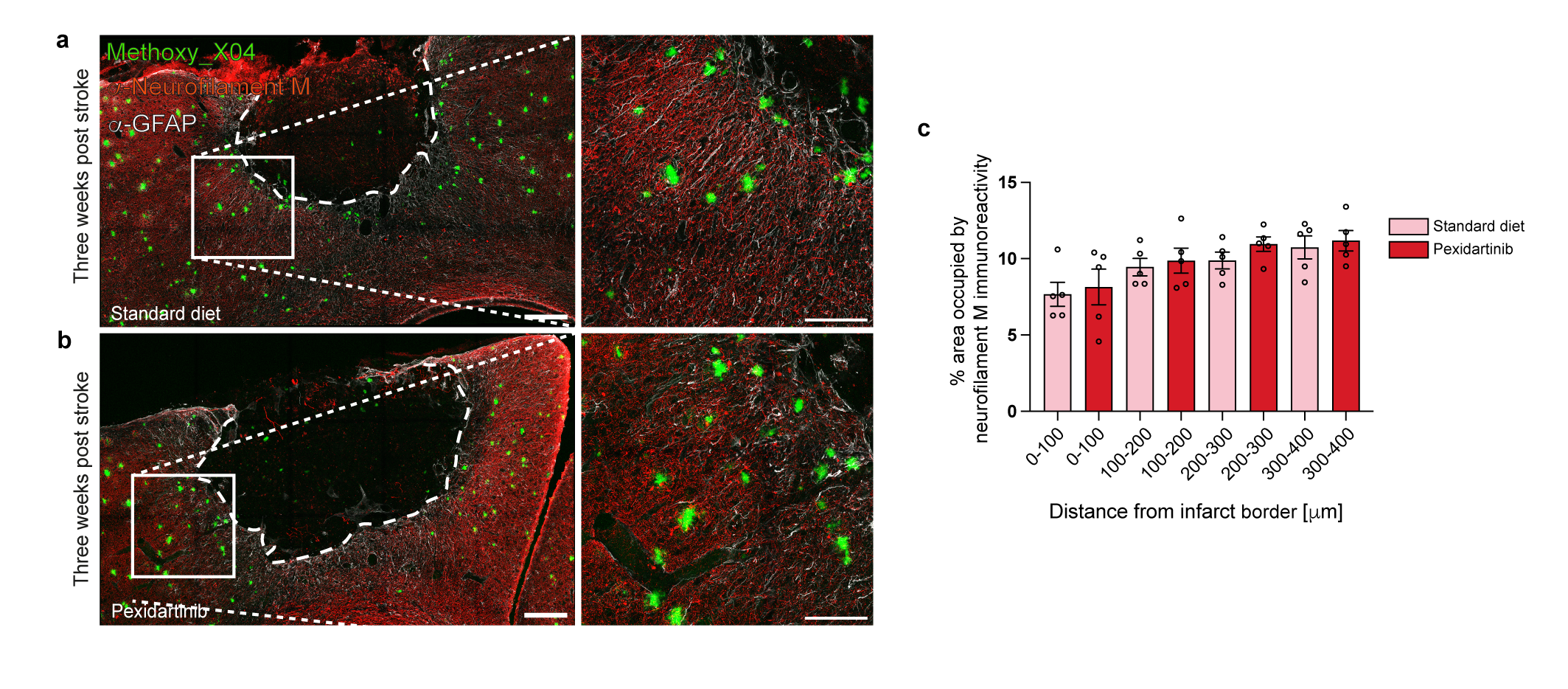


**Extended Data Figure 5. Neurofilament M-occupied area is not significantly different in the presence of pexidartinib.** (a) Representative images of neurofilament M immunoreactivity in the peri-infarct region three weeks post stroke from mice either on standard diet or (b) pexidartinib (n = 5 standard diet mice, 3 males 2 females, n = 5 pexidartinib-treated mice, 3 males 2 females). (c) No significant difference was found in the area covered by neurofilament M immunoreactivity between mice treated with pexidartinib or standard diet. Scale bar = 200 μm and 100 μm for insets. Ordinary one-way ANOVA with Holm-Šidák’s multiple comparison test (c).* = p < 0.05, ** = p <0.01, *** = p < 0.001, **** = p <0.0001. For full statistical details, see Supplementary Table 2.


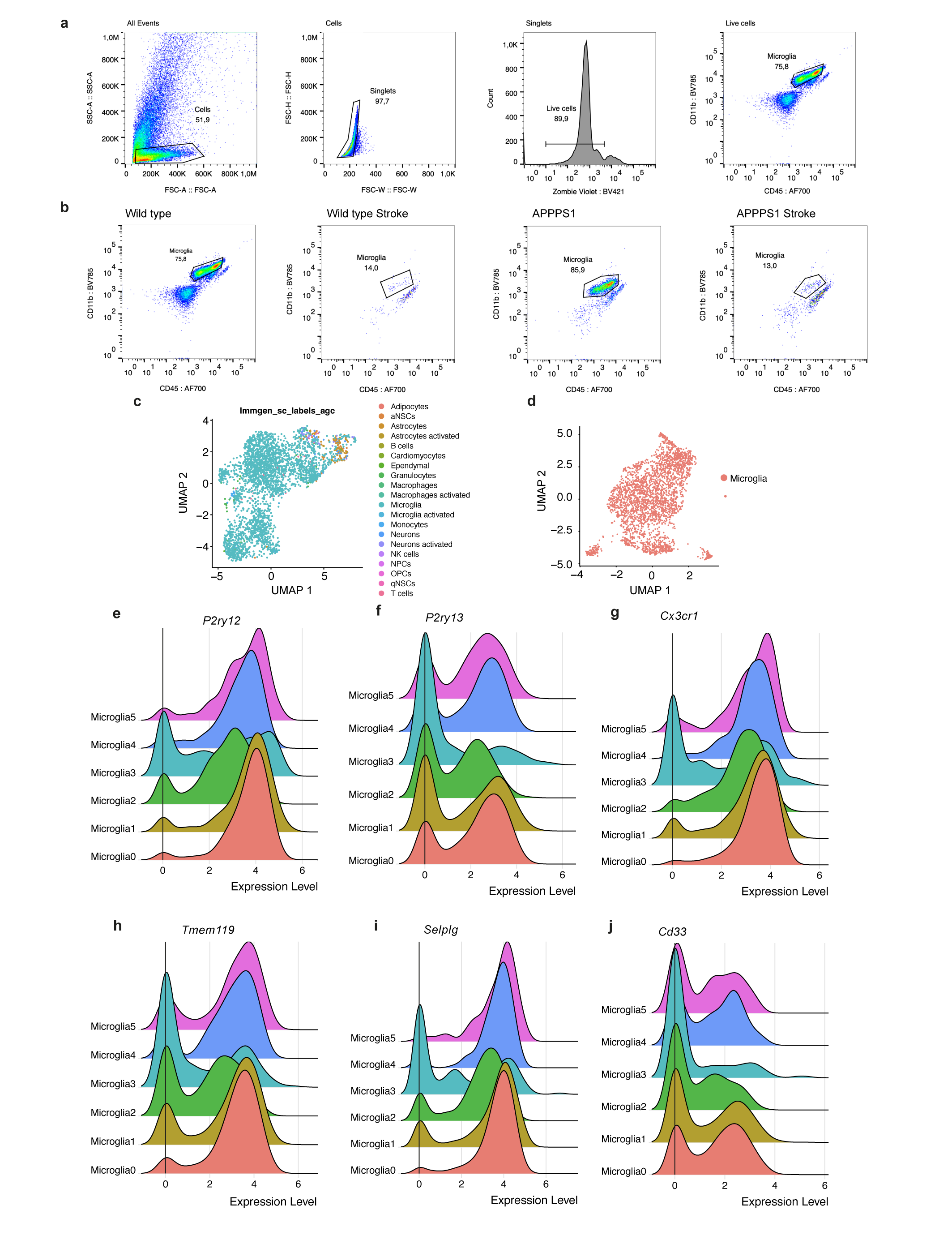


**Extended Data Figure 6. Validation of microglial sorting strategy.** (**a**) Representative snapshots illustrating the gating strategy utilized to FACS-sort microglia from (**b**) wildtype, wildtype stroke, APPPS1 and APPPS1 stroke mice. (**c**) UMAP illustrating the results of automatic cell type detection of FACS-sorted microglia prior and (**d**) after exclusion of non-microglial cells. (**e-j**) Ridge plots illustrating the expression level of microglia marker genes within each microglial cluster.


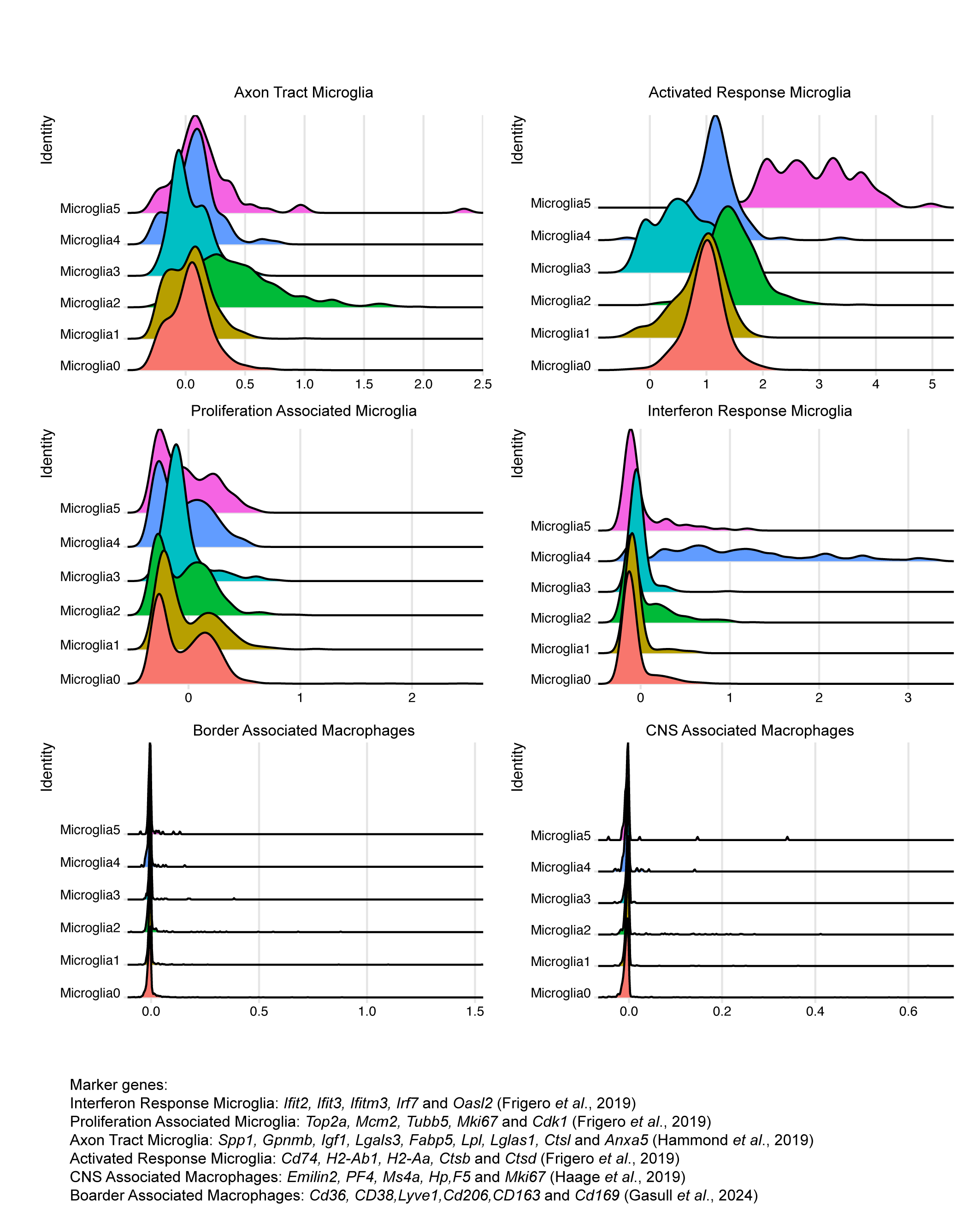


**Extended Data Figure 7. Gene expression profiles of known microglial subtypes.** Ridge plots illustrating the expression of marker genes of previously published microglial subtypes.


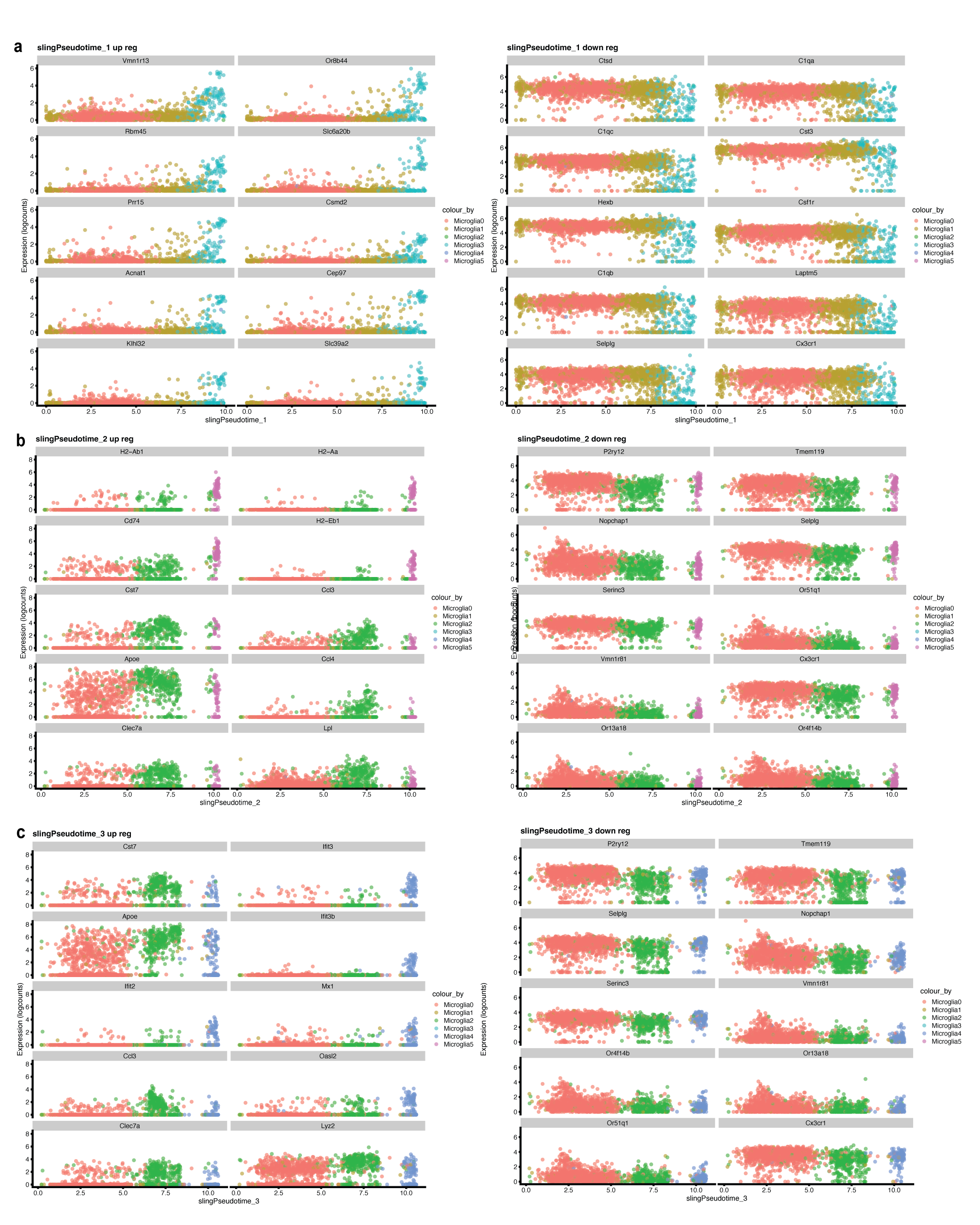


**Extended Data Figure 8. Marker genes determining different pseudotime trajectories.**  (**a**) Marker genes (upregulated left column, downregulated right column) for the pseudotime trajectories from homeostatic microglia 1 to metabolically-active microglia, (**b**) homeostatic microglia 1 to MHC-class II microglia and (**c­­­­­**) homeostatic microglia 1 to IRM.


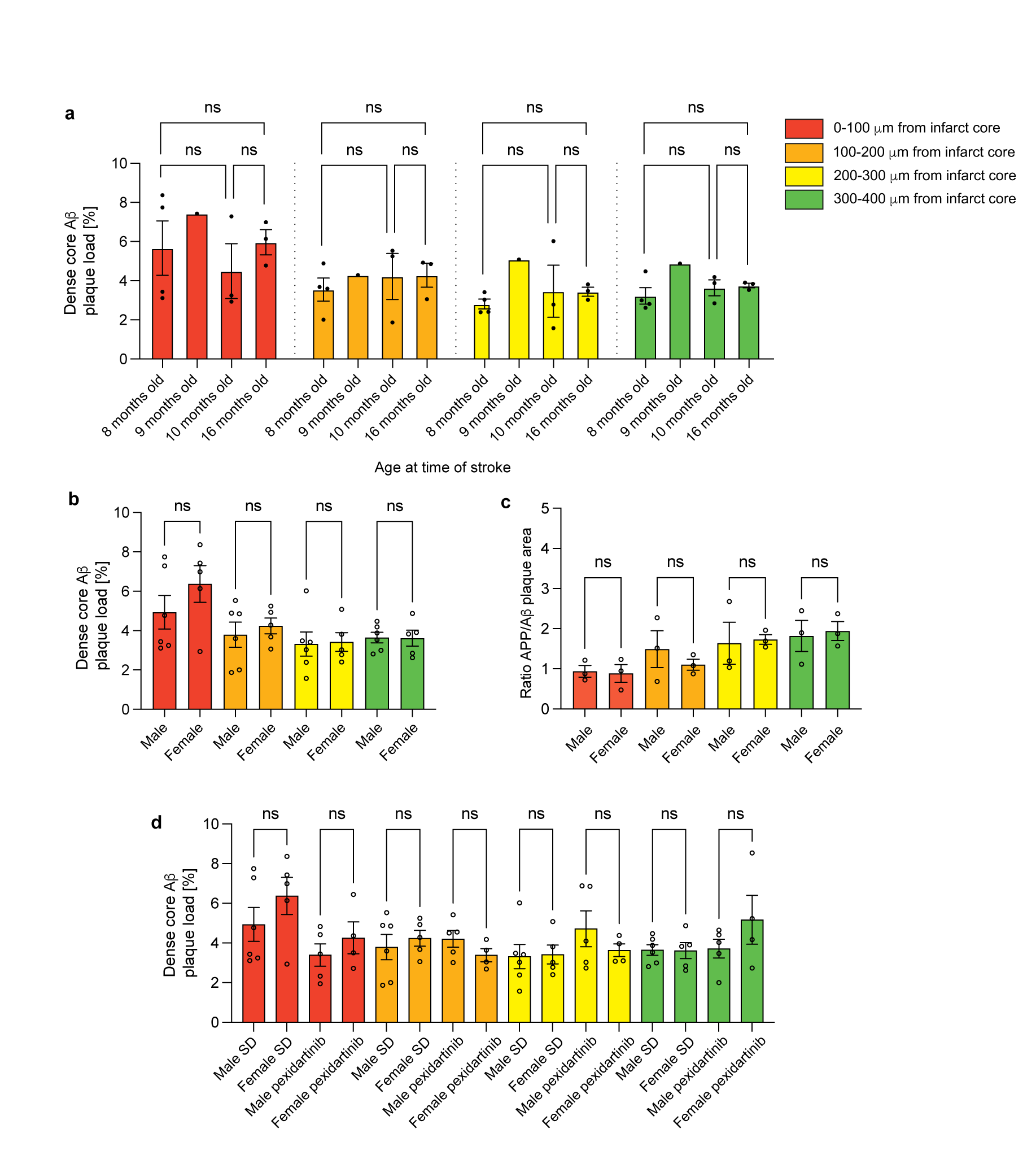


**Extended Data Figure 9. No sex or age differences were observed in the study. (a)** Inter-age comparison of dense core Aβ plaque load at three weeks post stroke suggests no age dependent phenotype. Note that only groups with n = 3 or higher were used for statistical analysis. **(b)** Similarly, no differences were observed in dense core Aβ plaque load, **(c)** the radio of APP to Aβ plaque area or **(d)** the dense core Aβ plaque load after pexidartinib treatment between males and females. Ordinary one-way ANOVA with Šidák’s multiple comparison test. * = p < 0.05, ** = p <0.01, *** = p < 0.001, **** = p <0.0001. For full statistical details, see Supplementary Table 2.
