# Supplementary material for: Ischemic injury triggers a protective microglial phenotype in models of Aβ pathology
Source: J Neuroinflammation. 2026 Jun 9;23:213. doi: 10.1186/s12974-026-03897-x (PMC13292430; doi:10.1186/s12974-026-03897-x)
Supplement: Supplementary file 8 — Supplementary Material 8. Methods [file 12974_2026_3897_MOESM8_ESM.docx]

**Methods**

**Mice (experimental animals)**

Animal experiments and husbandry were carried out in accordance with guidelines established by the animal welfare committee of the Johann Wolfgang Goethe-Universität Frankfurt am Main and in accordance with regulations established by the state of Hessen (Germany). Mice were kept under a standard light/dark cycle with access to food and water *ad libitum*. WT and hemizygous APPPS1^1^ transgenic mice (C57BL/6J-Tg(Thy1-APPSw,Thy1-PSEN1*L166P)21Jckr/J) harboring two transgenes under the control of the *Thy1* promoter (human APP^KM670/671NL^ and PS1^L166P^) were used in the study. Mice were kept on a C57BL/6J background. Since we observed no sex differences nor any phenotypic impact resulting from age differences after 5 months of age, a comparatively wide age range of 5 to 18 month old APPPS1 mice of both sexes were used in this study. Additionally, hemizygous APP23^2^ transgenic mice (harboring only the human APP^KM670/671NL^) mice were used but at a more advanced age (16-22 months) due to slower Aβ plaque kinetics in this model. Males and females were mixed for this study. Please see figure legends for n of each sex used in each experiment.

**Ischemic stroke**

Mice received an intraperitoneal (i.p.) injection of rose Bengal (Sigma) (100 mg/kg) and were anesthetized using 5% isoflurane. After the surgical anesthetic plane was reached, anesthesia was maintained at 2% isoflurane. A sagittal incision was made in the skin to expose the skull. The targeted brain region (somatosensory cortex, hindlimb) was irradiated through the skull for 20 minutes with 530 nm laser light (11 mW/cm^2^) resulting in thrombosis. Mice were administered a sub-cutaneous injection of carprofen (5 mg/kg) and the incision closed using 4/0 absorbable suture and Histoacryl. Mice were subsequently allowed to recover prior to returning to their home cages.

**Pexidartinib treatment**

To deplete brain resident microglia^3^, mice were treated with the drug pexidartinib (PLX-3397, HY-16749 Hyultec GmbH) that was administered via food at a concentration of 290 ppm (290 mg/kg) according to AIN-76A standard diet food of Research Diets Inc. (provided by Ssniff Spezialdiäten GmbH) for three consecutive weeks before the stroke-inducing surgery followed by three more weeks after the stroke.

**Transcardial perfusion and tissue freezing for histology**

Mice were sacrificed with an overdose of isoflurane and, shortly after death, transcardially perfused with 20 ml of room temperature (RT) phosphate-buffed saline (PBS) followed by 20 ml of ice-cold 4% paraformaldehyde (PFA). Brains were removed, post-fixed for two hours in 4% PFA at 4 °C and then transferred to 30% sucrose in PBS at 4 °C until sunk. Subsequently, brains were frozen in liquid nitrogen and stored at -20 °C until sectioning. 40 μm free-floating sections were prepared using a sliding microtome (Slee) and subsequently stored in freezing solution (30% glycerol, and 30% ethylene glycol in 1x PBS) at -20 °C until further use.

**Immunohistochemistry/histology**

Sections were washed 3x for 5 minutes with PBS and subsequently permeabilized and blocked with 0.5% triton X-100 in PBS and 5% donkey serum (DS) in PBS with 0.02% sodium azide, respectively, for one hour at RT. Sections were then incubated in primary antibodies (Supplementary Table 1) in PBS containing 0.5% λ-carrageenan (Sigma) and 0.02% sodium azide overnight at 4 °C. Subsequently, sections were washed 3x for 5 minutes with PBS with 0.05% tween-20 (PBST) and sequentially incubated with the appropriate secondary antibodies (Supplementary Table 1) in PBS containing 0.5% λ-carrageenan (Sigma) and 0.02% sodium azide at RT for two hours. Sections were then washed 3x for 5 minutes with PBST and transferred to PBS for mounting on Superfrost Plus microscopy slides and coverslipped with Fluoromount-G. For labelling of Aβ using methoxy_X04, a half hour incubation of 2% Methoxy_X04 (Tocris) in DMSO in PBS with 7.66% Kolliphore-EL (Sigma) was performed prior to final washes and mounting.

**Luminescent-conjugated oligothiophene labelling of Aβ *ex vivo***

hFTAA and qFTAA were kindly supplied by Prof. K. Peter R. Nilsson. Sections were washed 3x for 5 minutes with PBS and subsequently permeabilized and blocked with 0.5% triton X-100 in PBS and 5% DS in PBS with 0.02% sodium azide, respectively, for one hour at RT. Sections were then incubated in rabbit anti-GFAP (1:1000, DAKO) in PBS containing 0.5% λ-carrageenan (Sigma) and 0.02% sodium azide overnight at 4 °C. Sections were then washed 3x for 5 minutes with PBST and sequentially incubated with anti-rabbit IgG Alexa 647 (1:1000, Invitrogen)  in PBS containing 0.5% λ-carrageenan (Sigma) and 0.02% sodium azide at RT for two hours. Sections were then washed 3x for 5 minutes with PBST and then incubated with (3 μM) hFTAA for 30 minutes, washed 3x with PBST and then incubated with (6 μM) qFTAA for 30 minutes at RT. Sections were then washed 3x for 5 minutes with PBST and transferred to PBS for mounting on Superfrost Plus microscopy slides and coverslipped with Fluoromount-G.

**Luminescent-conjugated oligothiophene labelling of Aβ *in vivo***

Mice were administered an intraperitoneal injection (2 μl/g) of hFTAA in sterile saline (10 mg/ml) one day prior to transcardial perfusion for iDISCO experiments.

**iDISCO**

Whole-brain clearing was performed using only the “tissue-clearing” section of the previously described iDISCO protocol^4^ since Aβ labelling was already performed via *in vivo* injection of hFTAA. Briefly, samples were dehydrated in a gradient of methanol in PBS (20%, 40%, 60%, 80%, 100%, 100%) each for one hour on a tube rotator at RT. Delipidation was then performed by incubating the samples in 66% dichloromethane (DCM) with 33% methanol overnight at RT. The next day, samples were transferred to 100% DCM for 45 minutes, following by a 2-hour incubation in dibenzyl ether for clearing. Samples were then transferred into a brown glass vial filled with ethyl cinnamate until imaging. Prior to imaging, brains were transferred to custom 3D printed microscope slides featuring an approximately mouse-brain sized chamber filled with ethyl cinnamate. The chamber was then covered with a glass coverslip and permanently attached to the 3D printed microscope slide with epoxy.

**Wide-field and laser-scanning confocal microscopy**

Brain sections were imaged at an epifluorescence microscope (Imager M.1, Zeiss) using a 10x EC Plan-NEOFLUAR 10x/0.3 objective or a laser-scanning confocal microscope (SP8, Leica) using an HC FLUOTAR L 25x/0.95 NA water-immersion objective.

**Spectral scans**

For spectral scan analysis of Aβ around stroke lesions, images were taken at a Zeiss 2-photon microscope (Zeiss LSM 880) containing 32 photon multiplier tubes (PMTs) arranged specifically to detect different emission spectra of used luminescent conjugated oligothiophenes (LCOs), such as qFTAA and hFTAA, in a range of 410 – 695 nm in ~9 nm steps after excitation with a 2-photon laser (Mai Tai® DeepSee™) at 780 nm.

**2-photon microscopy of cleared mouse brains**

iDISCO-cleared mouse brains were imaged using a custom 2-photon microscope (based on a previously published design^5^) equipped with a Chameleon Ultra II laser (Coherent) and controlled by ScanImage software (MBF Biosciences). hFTAA was excited at 900 nm (laser power <50 mW). Emitted light was detected using a non-descanned Hamamatsu detector, a 560 nm dichroic mirror and a 605/70 nm bandpass filter. Z stacks (7 μm steps) were taken using a 10x EC Plan-NEOFLUAR 10x/0.3 objective

**Confocal Raman microscopy**

Raman imaging was performed in regions of interest with a confocal Raman microscope Witec 300R^+^ (Witec GmbH, Ulm, Germany) equipped with a 532 nm laser and a 50x objective (NA 0.8) and set to a power of 5 mW in front of the objective. Each scan was acquired in an area of 70x70 µm^2^ with a resolution of 0.333 µm and an integration time of 0.3 s. Stroked APPPS1 mice (1 scan 0-100 μm from the infarct border and 1 scan 300-400 μm from the infarct border) and APPPS1 control mice (1 scan each) were imaged and compared.

**Hyperspectral data processing**

Spectral background was subtracted using the ‘shape’ function (filter size: 100) and cosmic rays were removed in the Project FOUR software (Witec GmbH, Ulm, Germany). The data set was then imported to Matlab (The Matlab Inc., Natick, USA) for smoothing using a Savitzky-Golay-filter (window size: 9, order: 3) and normalization using the standard-normal-variate (SNV) method. Spectral unmixing was applied using vertex component analysis (VCA)^6^ the Raman Light App^7^ to identify spectral signatures of Aβ plaques in the tissue. For peak ratio analysis, the β-sheet peak (1238 cm-1) and phenylalanine peak (1005 cm-1) were divided by a peak with a constant intensity (1452 cm-1) in the Aβ spectral signature^8^.

**Data analysis**

All statistical analyses and the generation of graphs were performed using GraphPad Prism 9 software. Data analysis was performed using FIJI software for Aβ plaque, APP, Iba1, CD68, and spectral scan analysis. Microglia-Aβ plaque contact-area analysis was performed in tandem with FIJI and Imaris software (Imaris software (v 9.7), Oxford Instruments). Figures were prepared using Fiji, Bitplane Imaris and Adobe Illustrator.

**Spectral scan analysis**

For spectral scan analysis, a script was developed by Georgi Tushev from the Max-Planck Institute for Brain Research in Frankfurt am Main, Germany. For spectral scan analysis, Aβ plaques from different conditions were investigated. At least n = 5 Aβ plaques in different regions around stroke (0-400 μm) with varying time points after stroke were examined (1wps, 3wps, and 9wps), as well as the Aβ plaques of microglia-depleted brains that received a stroke (3wps PLX) and Aβ plaques from APPPS1 hemizygous control brains from different age groups (5, 9 and 16 months old).

qFTAA detects mature Aβ fibrils (dense core Aβ plaques), whereas hFTAA detects both mature Aβ and protofibrils^9,10^. ROIs of the Aβ signal were created depending on the emission peaks of qFTAA (502 nm) and hFTAA (588 nm)^11^ using an automated threshold of FIJI software (“Li dark”) and a minimum area of 25 μm² for each Aβ plaque. The ROIs created by the script for the dense-core Aβ plaques and the halos surrounding the Aβ plaques were then assigned to the different regions (0-400 μm) around the lesion. These ROIs were used to assess the Aβ load of dense-core Aβ plaques and diffuse, immature protofibrils by dividing the area of dense-core Aβ plaques and halo in the respective region around the stroke lesion (ratio of dense-core to halo). Furthermore, the maturation state of the Aβ plaques, depending on the signal intensity in each channel captured by the different PMTs, can be assessed. The signal intensity was captured in 32 channels from 410 nm to 695 nm, each covering a signal of around 9 nm of wavelength. The 32 spectra of every Aβ plaque were normalized to the maximum intensity value of this respective Aβ plaque. Afterward, the values for each channel of all Aβ plaques were averaged for each mouse and each region around the lesion.

Two ways of data presentation were chosen to compare the spectra of the conditions and time points in the regions around the lesion. One was to display the averages of all channels as a whole. For the other, the ratios of the peaks of qFTAA (502 nm) and hFTAA (588 nm) were calculated by dividing the value for the qFTAA peak from the value of the hFTAA peak. Statistics were performed using the ratios of the wavelengths.

Furthermore, images absent of stroke lesions (e.g., pictures from APPPS1 control brain sections) were analyzed as described above without any distance measurements. Notably, GFAP was used to identify the lesion border, which was detected with a secondary antibody conjugated to Alexa-647. Faint signals of this secondary antibody may be visible in the pictures and hence in the spectra of 647 nm. This signal, however, does not influence the ratio peak analyses of qFTAA and hFTAA.

**Image stitching**

Several pictures covering the lesion and peri-infarct region taken at the epifluorescence microscope required stitching to ensure a covered area of 400 μm tissue around the lesion in one image. This was accomplished using the 2D stitching plugin provided by FIJI software. When imaging with SP8 confocal microscope to cover sufficient tissue around the lesion, tile scans were performed, and the single images were subsequently stitched automatically to a merged image by the provided Leica LAS X software.

**Regions around lesion**

To analyze the peri-infarct region around the lesion for images taken at the epifluorescence microscope, the SP8 confocal microscope, or the Zeiss 880 2-photon microscope, the peri-infarct region was segmented into regions of 100 μm width starting from the delineation of the glial scar up to 400 μm distal from the lesion. Using FIJI software, along the border of the lesion, a ROI was manually drawn with the glial scar as a template. This ROI was enlarged by the respective size to get regions containing all the tissue for up to 400 μm. (lesion-100 μm, lesion-200 μm, lesion-300 μm and lesion-400 μm). The ROIs were then subtracted from each other appropriately to attain the desired region ROIs (0-100 μm, 100-200 μm, 200-300 μm, and 300-400 μm).

**Aβ plaque analysis**

Epifluorescence images were processed with FIJI software to analyze the Aβ plaques surrounding the lesion in the different regions or on control sections. The background of the Methoxy-X04 signal pictures was subtracted with the rolling ball method with 50 pixels, followed by blurring according to the Gaussian blur filter (sigma = 2). A threshold was applied according to the mean grey value of the image plus one standard deviation that was measured using FIJI excluding the infarct lesion. ROIs for the plaques were created using the “Analyze particles” function. Aβ plaques were selected manually depending on the distance from the lesion. With the information provided by the FIJI software, the Aβ plaque number/mm², Aβ plaque area/mm² (plaque load), and the average Aβ plaque size were calculated. For Aβ plaque analysis of control sections, all Aβ plaques visible in the field of view were considered (plaques touching the edges were excluded using the “analyze particles” function). Aβ plaque analysis was performed as described above to determine diffuse Aβ in control sections using Methoxy-X04 and hFTAA. The determined load for both dyes was subtracted from one another to attain the diffuse Aβ load.

**ApoE analysis**

8 bit maximum intensity projection images were used for this analysis. ROIs for regions around the lesion were performed as described above. ApoE intensities in the respective regions around the lesion were determined by measuring the mean grey value of ApoE immunoreactivity within the respective region around the lesion using FIJI software.

**APP analysis**

8-bit maximum projection pictures of the acquired merged SP8 confocal images were used for analysis. Aβ plaque analysis was performed similarly as described above using the pictures of the channel containing the Aβ plaque signal. A threshold with the mean grey value plus 2*standard deviation (values obtained excluding the lesion) was applied for the Aβ plaques. As only the total Aβ plaque area per region was needed, any signal of plaques ranging outside of one of the regions was cut out and removed to only receive the total Aβ plaque area per region. The Aβ plaque area per region around the lesion was automatically calculated by FIJI software.

The fluorescent background of images with APP signal was subtracted using the rolling ball method provided by FIJI software with a size of 50 pixels. Afterward, for analysis of the APP area / Aβ plaque area ratio, a threshold was applied with mean grey value plus 2*standard deviation of the whole image, excluding the infarct lesion. The area of the APP signal was measured in all regions using the “Analyze Particles” function, and the total APP area per total Aβ plaque area for the different regions was calculated.

**Iba1 analysis**

Aβ plaque load analysis was performed as described above on 8-bit maximum projection images acquired via confocal microscopy. The ROIs for the plaques were enlarged to obtain ROIs covering 15 μm around the plaques. All ROIs were saved for later analysis. The background of the channel containing the Iba1 signal was subtracted with the rolling ball method with a size of 50 pixels. All the Iba1 signal outside the region of interest and 15 μm around the Aβ plaques was removed only to analyze the microglia close to the Aβ plaques and in the respective region around the lesion. An automated threshold provided by FIJI (“Huang2”) was applied, and by using the “Analyze Particles” function, the covered area of the Iba1 signal around the Aβ plaques was determined.

**Microglia-Aβ plaque contact area analysis**

Before analyzing the microglia- Aβ plaque contact area with Imaris software, using FIJI software, the 16-bit z-stacks acquired from confocal imaging, additional channels containing the different regions around the lesion (0-400 μm) in different colors were created. These channels were later used to assign the Aβ plaques to the regions in Imaris.

Then, using Imaris software, surfaces were reconstructed using the Methoxy-X04-positive signal to create surfaces for Aβ plaques and the Iba1-positive signal to create surfaces for microglia. Aβ plaque surfaces were manually selected and deleted to leave only Aβ plaques from one region around the lesion per reconstructed Aβ plaque surface. Microglial surfaces were generated based on the Iba1 signal in the entire image. The area of Aβ plaque surfaces in contact with microglial surfaces was calculated using the “XTension” “Surface-Surface Contact Area”. For this, the Aβ plaque surfaces were selected as the primary surface and the microglial surface as the secondary surface.

**CD68 analysis**

For analyzing CD68 intensity in microglia close to Aβ plaques, Aβ plaque load analysis and Iba1 analysis were performed as described above. The ROIs for Iba1-positive microglia were saved for each region around the lesion. Using the 8-bit max projection pictures of the channel showing the CD68 signal and the ROIs acquired for the Iba1 positive signal, CD68 intensity (mean grey value) was measured in microglia near Aβ plaques using FIJI software.

**PU.1 analysis**

For analyzing PU.1 + nuclei in close proximity to Aβ plaques, a surface was created in Imaris for each channel (Methoxy_X04 and PU.1) in the peri-infarct regions of interest (i.e. 0-100 µm, 100-200 µm, 200-300 µm and 300-400 µm). A “distance to image border” filter of minimum 10 was set for the Methoxy_X04 surface to exclude Aβ plaques close to the image border. For the PU.1 surface, “spots” were created with an estimated XY diameter of 5 µm with background subtraction. Spots that were more than 50% outside of the image border of each ROI were excluded. The Xtension “find spots close to surface” with a threshold of 10 µm was subsequently used to detect PU.1 nuclei in close proximity to Aβ plaques.

**Neurofilament-M analysis**

The background of 8-bit maximum projections of confocal images containing the neurofilament-M signal was subtracted with the rolling ball method with 10 pixels, and subsequently, a threshold was applied according to the mean grey value of the whole image plus one standard deviation that was measured using FIJI not considering the infarct lesion. The total area of the present neurofilament-M signal was measured for each region up to 400 μm around the lesion using the “Analyze Particles” function.

**LAMP1 analysis**

8-bit maximum projections of SP8 confocal Z-stack images were used for analysis. Aβ plaque analysis was performed in FIJI as described above. A gaussian blur with a sigma of 2 was then applied to the LAMP1 channel. For analysis of the LAMP1 area, a threshold was applied with mean grey value plus 2*standard deviation of the whole image, excluding the infarct lesion. ROIs of Aβ plaques were expanded by 20 μm to analyze Aβ plaque-associated LAMP1 immunoreactivity The percentage of area occupied by LAMP1 immunoreactivity was measured within the expanded Aβ plaque ROIs for the different regions using the “measure” function.

**Intra/extra neuronal hFTAA signal segmentation**

8-bit maximum projection pictures of SP8 confocal Z-stack images were taken of three weeks post stroke brain sections labelled with Neurotrace 500/525 (ThermoFisher, N21480), hFTAA and Methoxy_X04. The following channels were aquired; Methoxy_X04 in blue, neurotrace, hFTAA and methoxy_X04 in green and hFTAA alone in red. Surfaces were generated in Imaris for Methoxy_X04 and hFTAA. Due to the overlapping emission spectra of neurotrace, hFTAA and Methoxy_X04 in green, the hFTAA surface was used as a mask to set all voxels of the neurotrace/hFTAA/Methoxy_X04 channel within the hFTAA surface to zero; thus generating a new channel with only neurotrace signal (i.e. removing Aβ plaques from this channel). A surface was then generated of the isolated neurotrace signal which was used as a mask on the hFTAA channel to set voxels outside to zero to create an intraneuronal hFTAA channel. This was repeated again, setting the voxels inside to zero to create an extraneuronal hFTAA channel. However, this extraneuronal hFTAA channel still contains Methoxy_X04+ dense core Aβ plaques (which are also hFTAA positive). Therefore it was necessary to remove the Methoxy_X04 labelled Aβ signal from the extraneuronal hFTAA channel to attain a new channel containing Aβ that is exclusively labelled with hFTAA. Therefore, the surface of the Methoxy_X04 channel was used as a mask to set inside voxels of the extracellular hFTAA channel to zero resulting in an extraneuronal hFTAA channel without methoxy_X04+ dense core Aβ plaques. Surfaces were then generated of the extraneuronal hFTAA channel minus methoxy_X04 and the intraneuronal hFTAA channel to attain the total volume of extraneuronal and intraneuronal hFTAA within the image.

**Statistical analysis**

Statistical analyses were performed using the software Prism 9 (GraphPad Software). In general, for all the conducted experiments and analyses, when comparing the results for the regions around the lesion (0-400 μm) within one condition (1wps, 3wps, 9wps, or 3wps pexidartinib), they were evaluated using a repeated-measure one-way ANOVA Tukey’s multiple comparisons test unless otherwise stated. There, sphericity was assumed, and the mean of each region was compared to the mean of every other region. A mixed-effect analysis was performed if values were missing for the repeated measures analysis.

For multiple comparisons between the different conditions (for example, comparing 0-100 μm region around the stroke between 1wps, 3wps, 9wps, and ctrl; or comparing the different age groups (5, 9, 16 months) of control sections) an ordinary one-way ANOVA Tukey’s multiple comparisons test was performed. For comparisons of the four regions around the lesion of 3 wps standard diet to 3 wps pexidartinib treatment, an ordinary one-way ANOVA Šídák’s multiple comparisons test was performed with selected comparisons only to compare the respective regions (e.g., 0-100 μm SD vs 0-100 μm PLX). Possible outliers were identified using the “Identify Outliers” function of Prism 9 using the ROUT method. Cleaned data were then further processed as described above. Unless otherwise stated, graphs display the individual values and the mean ± standard error mean. For details regarding statistical tests see Supplementary Table 2.

**scRNA-seq**

Mice received a photothrombotic stroke three weeks before microglial isolation. Mice that suffered from a photothrombotic stroke and control APPPS1 and wild-type animals were deeply anesthetized with ketamine/xylazine solution and perfused with ice-cold 1x PBS. The brains were cut out, and the right cortex was dissected. The stroke lesion was dissected, including a small amount of tissue surrounding the lesion. The control animals' whole cortex of the right hemisphere was dissected.

The dissected tissue was homogenized in a petri dish covered with 500 μl dissection medium (0.5 % D-Glucose and 0.1 mg/ml DNase I in 1x HBSS w/o Ca+Mg) by mincing it into small pieces with a scalpel. The tissue was transferred into a 7 ml capacity Dounce homogenizer along with a 1 ml ice-cold dissection medium. The solution containing tissue (around 2 ml total volume) was slowly homogenized five to six times and transferred into a 5 ml Dounce homogenizer, followed by three additional slow homogenization steps. The solution was transferred through a 70 μm cell strainer into a 15 ml Falcon® tube, and the cell strainer was washed with 1x HBSS and filled up to 15 ml total volume. Afterward, it was centrifuged for 20 minutes at 300 G at 4 °C, and no brake was set in the centrifuge.

4 ml of 30 % isotonic Percoll in HBSS was added into a fresh 15 ml Falcon tube and underlaid with 4 ml 37 % Percoll, additionally dyed with Phenol red to differentiate the different Percoll gradients. The supernatant of the tissue solution was discarded, and the cell pellet was resuspended in 5 ml 70 % Percoll and put underneath the 37 % Percoll using a long glass Pasteur pipette. The solution was centrifuged at 800 G and 4 °C for 30 minutes with no brakes set in the centrifuge. After centrifugation, the myelin layer was first carefully removed from the top of the gradient. The cells forming a white halo from the interphase between 37 % and 70 % gradients were carefully collected and transferred into a new 15 ml Falcon tube that was then filled with FACS buffer (10 mM EDTA, 5 mM HEPES and 2% FCS in 1x HBSS w/o Ca + Mg). The tube was inverted and flicked to dissolve possible remaining high-density Percoll at the bottom of the tube and centrifuged at 300 G and 4 °C for 20 minutes. Afterward, the supernatant was discarded, and the cell pellet was resuspended in 200 μl FACS buffer and transferred into a 1.5 ml Eppendorf® cup. 0.5 μl Fc block (1:500, BD, Art. No. 553141) was added to the solution and incubated for 10 minutes at 4 °C on a rotation wheel. It was washed with 1 ml FACS buffer and centrifuged at 300 G and 4 °C for five minutes with the brake off. The supernatant was discarded and ideally reduced to a total volume of around 100 μl. Then, 0.5 μl of each CD11b – BV785 (1:200, Biolegend, Art.No. 101243) and CD45 – AF700 antibody (1:200, Biolegend, Art.No. 103128)was added to the solution and incubated for 15 minutes at 4 °C on the rotation wheel in the dark. The solution was washed again with 1 ml FACS buffer and centrifuged for five minutes at 300 G and 4 °C with brakes off. The supernatant was discarded and filled up to 250 μl with FACS buffer.

With a cell sorter (Sony SH800S cell sorter), single cells positive for CD11b and CD45 were sorted into 384-well plates containing lysis buffer and oligonucleotides for Smart-seq2^12^ and stored at -80 °C until further processing.

Sorted cells were further processed using an adopted Smart-seq2 protocol for 384-well plates^13^. In short, 384-well plates containing single cells were thawed and cells were lysed and mRNA denatured by incubating the plates at 95°C for 3 minutes and put back on ice. Subsequently, 2.7 μl of 440 first-strand cDNA reagent mix, containing 1x First-strand buffer, 5 mM DTT, 1 M betaine, 14 mM MgCl2, 5 U RNase inhibitor (Takara), 25 U Superscript II Reverse Transcriptase, and 1 μM Template-Switching oligonucleotides in nuclease-free water, was added to each well. Plates were incubated at 42°C for 90 minutes, 70°C for 15 minutes, and kept on hold at 4°C. Afterward, 7.5 μl of the pre-amplification mix, consisting of 1x KAPA HiFi HotStart Readymix and 0.12 μM ISPCR primers in nuclease-free water, was added to each well. Plates were then incubated as followed: 98°C for 3 minutes, 23 cycles of 98°C for 20 seconds; 67°C for 15 seconds; and 72°C for 6 minutes, then at 72°C for 5 minutes and kept on hold at 4°C. Purification of the resulting cDNA was performed using SeraMag SpeedBeads containing 19% w/v PEG 8,000 and a 1:0.8 ratio of cDNA/beads was used for cDNA precipitation. Purified cDNA was eluted in 14 μl of nuclease-free water. The quality of cDNA was randomly checked in 5% of the wells using the Tapestation High-Sensitivity D5000 assay. For the tagmentation reaction, 0.5 μl of 50-150 pg cDNA was mixed with a 1.5 μl Tagmentation mix containing 1x Tagment DNA buffer and Amplicon Tagment mix (Nextera XT DNA sample preparation kit). Plates were incubated at 55°C for 8 minutes and kept on hold at 4°C. 0.5 μl Neutralize Tagment buffer was added and incubated at room temperature for 5 minutes to inactivate Tn5. PCR amplification of adapter-ligated cDNA fragments was performed in a final volume of 5 μl containing the two index primers (Nextera XT Index kit) in a 1:5 dilution and Nextera PCR master mix. Plates were then incubated as followed: 72°C for 3 minutes, 95°C for 30 seconds, then 14-15 cycles of 95°C for 10 seconds; 55°C for 30 seconds; and 72°C for 30 seconds, then 72°C for 5 minutes and kept on hold at 4°C. 384 wells (the entire plate, including negative controls) were pooled in a single 2 ml tube (Eppendorf). Purification of cDNA was performed by adding 400 μl of cDNA in a 1:1 ratio with SeraMag SpeedBeads in 19% w/v PEG 8,000 in a 1.5 ml LoBind tube. Beads were washed with 1 ml of 80% ethanol. Purified cDNA was eluted in 200 μl of nuclease-free water. The concentration of the library was measured by Qubit according to the manufacturer’s instructions. The size distribution of the library was measured using the Tapestation High-Sensitivity D1000 or D5000 assay. The cDNA library pool was stored at -20°C until ready for sequencing.

The cDNA library pool was diluted to 2 nM and prepared for sequencing using the NextSeq 500 High Output Kit v2 or NovaSeq 6000 SP reagent kit 1.5 (75 cycles) according to the manufacturer’s instructions. Sequencing was performed on an Illumina NextSeq 500 or Illumina NovaSeq 6000 instrument. Preprocessing of the data was performed as previously described^13^.

Analysis was performed using the Seurat package and Harmony pipeline with R software. For the detailed cluster analysis we excluded any transcripts coded on either of the sex chromosomes, or do not have any canonical name yet (i.e. cDNAs from the RIKEN project or Gm* Genes). Samples were integrated across batches based on the 2000 most variable genes using the integrated anchoring approach as implemented in Seurat. Reads were Normalized and SCT-Transformed. UMAP dimensions were calculated based on the first 20 dimensions of the Harmony data reduction (selection based on elbow criteria of the skeeplot). Initial clustering was performed with a resolution of 0.6. Cell Cycle scoring was based on tinyatlas mouse gene data (https://github.com/hbc/tinyatlas). For identification of cell types and confirmation of microglial identity was based on a publicly available scRNA data^14^ and implemented in the celdex package. For any further analyses only cells labelled as Microglia were included. After gene and cell filtering, reclustering across all samples was performed including optimization for the clustering resolution (i.e. 0.6): A range from 0.1 to 1.2 resolution was implemented and best clustering was selected based on the most stable condition using clustree plots. A total of 6 clusters (Microglia 0-5 was identified). DAM scores and Homeostatic scores were calculated (AddModuleScore) on gene-sets previously published^15^. Scores were distributed bimodally and the cutoff for DAM+ or Homeo+ cells was manually set at the lowest point of inflection between the two peaks. Differences in cell distribution were tested using chi-square tests, and pairwise chi-square test with post hoc correction. For identifying markers and differentially expressed genes between two conditions the FindMarkers function with the “LR” method was applied, adjusted p-values are reported. GO-term enrichment analysis was done with the profiler package with the universe of all genes detected in the scRNA sample as background reference and the SCS-significance threshold as published^16^. Pseudotime analysis was performed using the slingshot packages with Microglia 2 as the starting cluster and default conditions. TSCANs function testPseudotime identified Genes differentially expressed across trajectories.

**Brain collection for spatial transcriptomics**

Mice were sacrificed three weeks post-stroke by cervical dislocation, rapidly decapitated and the brain removed and rolled on a tissue paper (VWR) to remove excess blood. The brain was then transferred to a sterile Petri dish and cut into two hemispheres using a new glass coverslip. Each hemisphere was then transferred to a Peel-a-Way paraffin embedding mold filled with Optimal Cutting Temperature (OCT, Leica Biosystems) compound (from a freshly-opened bottle). The embedding molds were then transferred to a Coplin jar filled with isopentane, which was then placed in a box containing dry ice and ethanol. To determine the RIN of each brain sample, 10x 10 μm coronal sections were taken from the OCT block containing the contralateral hemisphere using a freezing microtome (Leica Biosystems), whereas the OCT blocks containing each ipsilateral side were used exclusively for spatial transcriptomics.

**RNA integrity number (RIN) check**

The RNA was extracted using RNeasy Mini Kit (Qiagen, USA) according to the manufacturer’s protocol. The quality of the tissue was then checked by calculating its RNA Integrity Number (RIN), using the Agilent RNA 6000 Nano Assay kit with a 2100 Bioanalyzer (Agilent, USA), following the manufacturer’s protocol, as suggested by the Visium Spatial Tissue Optimization User Guide (CG000240 Rev E, 10x Genomics). Samples only with a RIN ≥ 7 were eligible for the transcriptomics experiments.

**Spatial transcriptomics**

Spatial transcriptomics were carried out using the Visium platform (10x Genomics) following the manufacturer’s instructions. Briefly, the slide was processed by following the methanol fixation, Immunofluorescence Staining & Imaging for Visium Spatial Protocols (CG000312, 10x Genomics). Briefly, the Visium Spatial Gene Expression slide was fixed, blocked, and then stained with primary antibodies for a total of 30 minutes at room temperature, washed five times, stained with secondary antibodies for 30 minutes at room temperature, and then washed another five times. Each APPPS1+stroke section was stained with a different set of antibodies; (1) Primary antibodies: rabbit CN6, goat anti-podocalyxin, rabbit anti-GFAP. Secondary antibodies: donkey anti-rabbit 488, donkey anti-goat 647, donkey anti-rabbit 555, donkey anti-goat 647. (2) rat anti-CD45, rabbit CN6, goat anti-GFAP. Secondary antibodies: donkey anti-rat 488, donkey anti-rabbit 555, donkey anti-goat 647. (3) rat anti-CD45, rabbit CN6, goat anti-podocalyxin. Secondary antibodies: donkey anti-rat 488, donkey anti-rabbit 555, donkey anti-goat 647. All sections were additionally stained with DAPI. The slide was mounted using RNase-free glycerol, and imaged using Zeiss Cell Observer Z.1 microscope. After imaging, the slide was further processed by following the Visium Spatial Gene Expression Reagent Kits - User Guide (CG000239, 10x Genomics).

RNA was extracted according to manufacturer’s instructions (Visium Spatial Gene Expression Slide & Reagent Kit, 16 rxns PN-1000184).The Library preparation was conducted according to the manufacturer indications (Visium Spatial Gene Expression Slide & Reagent Kit, 16 rxns PN-1000184).

The Raw reads were aligned against the mouse genome (mm10) and counted by StarSolo^17^ followed by secondary analysis in Annotated Data Format. Preprocessed counts were further analyzed using Scanpy^18^. Basic data quality control was conducted by taking the number of detected genes and mitochondrial content into consideration as well as coverage by tissue on spots. We removed 10502 data points in total that did not express more than 1000 genes or had a mitochondrial content greater than 30% and were not covered by tissue. Furthermore, we filtered 39771 genes if they were detected in less than 30 cells (<0.01%). Raw counts per cell were normalized to the median count over all cells and transformed into log space to stabilize variance. We initially reduced dimensionality of the dataset using PCA, retaining 50 principal components. Subsequent steps, like low-dimensional UMAP embedding and cell clustering via community detection, were based on the initial PCA.

Final data visualization was done using the CZ CELLxGENE Annotate package in combination with the CellxGene VIP extension^19^.

**Human brain section processing and analysis**

Human brain tissue was accessed from the Neurobiobank Munich. We deeply thank all brain donors and their families for facilitating this research. We are also very thankful to all current and former colleagues of the Neurobiobank Munich for their elaborate organization, processing, and diagnostics. Mounted human brain sections were heated at 58°C for 1 h, then deparaffinized and rehydrated (3 x 10 min Xylol, 3 x 5 min 100% EtOH, 2 x 5 min 96% EtOH, 1 x 5 min 70% EtOH, 1 x 5 min ddH2O)using standard protocols. For antigen retrieval, sections were boiled in citrate buffer (1.8 mM citric acid, 8.2 mM trisodium citrate, pH 6) at 90°C for 30 min. Nuclear staining was then performed by incubation with nuclear fast red (Morphisto, Art. No. 10264), according to manufacturer’s instructions. Subsequently, non-specific antibody binding was blocked by incubation with 5% normal donkey serum (in 0.3% Triton X-100 in PBS) for 1 h, followed by primary antibody incubation (goat anti-Iba1, 1:600, Novus, Art. Number NB100-1028) in 2% serum (in 0.3% Triton X-100 in PBS), over two nights at 4°C. After washing, the sections were incubated with the secondary antibody (donkey anti-goat, AF647-conjugated, Jackson Immuno Research, Art. Number 705­-605­-147, 1:250 in PBS) for 2 h at RT. For the LCO staining, sections were incubated with qFTAA (2.4 µM in PBS) and hFTAA (0.77 µM in PBS), according to standard protocols^11^. To include a fluorescent nuclear stain, propidium iodide (Abcam, 1:100) was added to the LCO staining solution and co-incubated for the entire duration. Subsequently, sections were incubated with TrueBlack Quencher (Biotium, 1:20 in 70% ethanol) for 10 s, according to manufacturer’s instructions, in order to reduce autofluorescence caused by lipofuscin and other tissue components. The sections were then dried at RT for 20 min and coverslipped with fluorescence mounting medium (DAKO), Art. No. S302380-2). The slides were dried overnight at RT and stored at 4°C until imaging.

To obtain an overview of overall tissue integrity and the distribution of ischemic neurons, one neighboring brain section per patient was stained with cresyl violet (Morphisto), according to standard protocols. Confocal imaging was performed with a Stellaris 5 microscope (Leica). All Aβ plaques identified throughout the entire human brain sections (based on hFTAA signal) were imaged and 30 µm Z-stacks were acquired with a 0.5 µm step size. Images were acquired with a HC PL APO CS22 40x/1.3 OIL objective and exported with a bit depth of 8. Using Imaris (Bitplane, V.10.2)., 3D surface reconstructions were created for every channel based on a fixed intensity threshold. Microglia were reconstructed using Iba1 signal, and Aβ plaques were reconstructed based on hFTAA (total Aβ plaque) and qFTAA (Aβ plaque core) signals, respectively. Reconstructed microglia were automatically categorized into “plaque-associated” and “plaque-distant” microglia, based on the smallest distance to an Aβ plaque (< 13 µm or ≧ 13 µm, respectively). Brightfield images were obtained with a SLIDEVIEW VS200 slide scanner (Olympus) at 20x magnification. Imaris data post-processing was performed in R Studio (V. 2024.12.0+467). Slide scanner images were analyzed and exported using OlyVIA software (Olympus, V.3.8).

**Supplementary references**

1. Radde, R. *et al.* Abeta42-driven cerebral amyloidosis in transgenic mice reveals early and robust pathology. *EMBO Rep* **7**, 940–946 (2006).

2. Sturchler-Pierrat, C. *et al.* Two amyloid precursor protein transgenic mouse models with Alzheimer disease-like pathology. *Proc Natl Acad Sci U S A* **94**, 13287–13292 (1997).

3. Spangenberg, E. *et al.* Sustained microglial depletion with CSF1R inhibitor impairs parenchymal plaque development in an Alzheimer’s disease model. *Nat Commun* **10**, 3758 (2019).

4. Manfredi-Lozano, M. *et al.* GnRH replacement rescues cognition in Down syndrome. *Science* **377**, eabq4515 (2022).

5. Rosenegger, D. G., Tran, C. H. T., LeDue, J., Zhou, N. & Gordon, G. R. A high performance, cost-effective, open-source microscope for scanning two-photon microscopy that is modular and readily adaptable. *PLoS One* **9**, e110475 (2014).

6. Nascimento, J. M. P. & Dias, J. M. B. Vertex Component Analysis: A~Fast Algorithm to Extract Endmembers Spectra from Hyperspectral Data. in *Pattern Recognition and Image Analysis* (eds. Perales, F. J., Campilho, A. J. C., de la Blanca, N. P. & Sanfeliu, A.) 626–635 (Springer, Berlin, Heidelberg, 2003). doi:10.1007/978-3-540-44871-6_73.

7. Schmidt, R. W., Woutersen, S. & Ariese, F. RamanLIGHT—a graphical user-friendly tool for pre-processing and unmixing hyperspectral Raman spectroscopy images. *J. Opt.* **24**, 064011 (2022).

8. Movasaghi, Z., Rehman, S. & Rehman, I. U. Raman Spectroscopy of Biological Tissues. *Applied Spectroscopy Reviews* **42**, 493–541 (2007).

9. Ellingsen, P. G., Nyström, S., Reitan, N. K. & Lindgren, M. Spectral correlation analysis of amyloid β plaque inhomogeneity from double staining experiments. *J Biomed Opt* **18**, 101313 (2013).

10. Nyström, S. *et al.* Evidence for age-dependent in vivo conformational rearrangement within Aβ amyloid deposits. *ACS Chem Biol* **8**, 1128–1133 (2013).

11. Rasmussen, J. *et al.* Amyloid polymorphisms constitute distinct clouds of conformational variants in different etiological subtypes of Alzheimer’s disease. *Proc Natl Acad Sci U S A* **114**, 13018–13023 (2017).

12. Picelli, S. *et al*. Smart-seq2 for sensitive full-length transcriptome profiling in single cells. *Nature methods* **10**, 1096-1098 (2013).

13. Verstegen NJM. *et al*. Single-cell analysis reveals dynamics of human B cell differentiation and identifies novel B and antibody-secreting cell intermediates*. Elife* **12**, e83578 (2023).

14. Benayoun, B. A. *et al.* Remodeling of epigenome and transcriptome landscapes with aging in mice reveals widespread induction of inflammatory responses. *Genome Res* **29**, 697–709 (2019).

15. Keren-Shaul, H. *et al.* A Unique Microglia Type Associated with Restricting Development of Alzheimer’s Disease. *Cell* **169**, 1276-1290.e17 (2017).

16. Kolberg, L. *et al.* g:Profiler—interoperable web service for functional enrichment analysis and gene identifier mapping (2023 update). *Nucleic Acids Research* **51**, W207–W212 (2023).

17. Dobin, A. *et al.* STAR: ultrafast universal RNA-seq aligner. *Bioinformatics* **29**, 15–21 (2013).

18. Wolf, F. A., Angerer, P. & Theis, F. J. SCANPY: large-scale single-cell gene expression data analysis. *Genome Biology* **19**, 15 (2018).

19. Li, K. *et al.* Cellxgene VIP unleashes full power of interactive visualization and integrative analysis of scRNA-seq, spatial transcriptomics, and multiome data. 2020.08.28.270652 Preprint at https://doi.org/10.1101/2020.08.28.270652 (2022).
